# Cortical lesions impact cognitive decline in multiple sclerosis via volume loss of nonlesional cortex

**DOI:** 10.1002/acn3.52261

**Published:** 2024-12-27

**Authors:** Eva A. Krijnen, Maureen van Dam, Albulena Bajrami, Piet M. Bouman, Samantha Noteboom, Frederik Barkhof, Bernard M.J. Uitdehaag, Martijn D. Steenwijk, Eric C. Klawiter, Ismail Koubiyr, Menno M. Schoonheim

**Affiliations:** ^1^ MS Center Amsterdam, Department of Anatomy and Neurosciences, Amsterdam Neuroscience Vrije Universiteit Amsterdam Amsterdam The Netherlands; ^2^ Department of Neurology Massachusetts General Hospital, Harvard Medical School Boston Massachusetts USA; ^3^ Institute of Psychology, Department of Health, Medical and Neuropsychology Leiden University Leiden The Netherlands; ^4^ Division of Neurology, Department of Emergency “S. Chiara” Hospital, Azienda Provinciale per i Servizi Sanitari (APSS) Trento Italy; ^5^ MS Center Amsterdam, Department of Radiology and Nuclear Medicine, Amsterdam Neuroscience Vrije Universiteit Amsterdam Amsterdam The Netherlands; ^6^ Queen Square Institute of Neurology and Centre for Medical Image Computing University College London London UK; ^7^ MS Center Amsterdam, Department of Neurology, Amsterdam Neuroscience Vrije Universiteit Amsterdam Amsterdam The Netherlands

## Abstract

**Objective:**

To assess the interrelationship between cortical lesions and cortical thinning and volume loss in people with multiple sclerosis within cortical networks, and how this relates to future cognition.

**Methods:**

In this longitudinal study, 230 people with multiple sclerosis and 60 healthy controls underwent 3 Tesla MRI at baseline and neuropsychological assessment at baseline and 5‐year follow‐up. Cortical regions (*N* = 212) were divided into seven functional networks. Regions were defined as either lesional or normal‐appearing cortex based on presence of a cortical lesion on artificial intelligence‐generated double inversion‐recovery scans. Cortical volume and thickness were determined within lesional or normal‐appearing cortex.

**Results:**

Prevalence of at least one cortical lesion was highest in the limbic (73%) followed by the default mode network (70.9%). Multiple sclerosis‐related cortical thinning was more pronounced in lesional (mean *Z*‐score = 0.70 ± 0.84) compared to normal‐appearing cortex (−0.45 ± 0.60; *P* < 0.001) in all, except sensorimotor, networks. Cognitive dysfunction, particularly of verbal memory, visuospatial memory, and inhibition, at follow‐up was best predicted by baseline network volume of normal‐appearing cortex of the default mode network [*B* (95% CI) = 0.31 (0.18; 0.43), *P* < 0.001]. Mediation analysis showed that the effect of cortical lesions on future cognition was mediated by volume loss of the normal‐appearing instead of lesional cortex, independent of white matter lesion volume.

**Interpretation:**

Multiple sclerosis‐related cortical thinning was worse in lesional compared to normal‐appearing cortex, while volume loss of normal‐appearing cortex was most predictive of subsequent cognitive decline, particularly in the default mode network. Mediation analyses indicate that cortical lesions impact cognitive decline plausibly by inducing atrophy, rather than through a direct effect.

## Introduction

Cognitive impairment is acknowledged as a prevalent and debilitating symptom in people with multiple sclerosis (MS), occurring in up to 65% of patients.^1^ Even though extensive tissue damage is present in the white matter (WM), damage to the gray matter (GM) has a stronger association with MS cognitive dysfunction.^1^ In particular, of the MR measures investigated, GM volume loss seems to be the most promising marker for overall cognitive impairment and is driven in part by neuroaxonal degeneration in the GM.^1^, ^2^ The specific structural and functional substrates of GM volume loss and their temporal association with neuroinflammatory pathology, however, are still under investigation.

GM neurodegeneration presumably begins at disease onset alongside neuroinflammation^3^, ^4^ and occurs not only at sites of overt demyelination, that is, cortical lesions (CLs), but also throughout normal‐appearing tissue without evidence of focal inflammation.^5^, ^6^ CLs have been found to relate to cognitive disability accumulation,^7^, ^8^ especially in visuospatial memory and processing speed,^2^ and even independently from cortical atrophy.^7^, ^9^ Some previous studies actually suggested CLs as possible substrates of cortical volume loss,^10^, ^11^ though others have associated cortical atrophy to WM pathology^9^, ^12^ and found cortical demyelination to rarely co‐localize with cortical atrophy,^13^, ^14^ suggesting only a minor role of CLs in the development of cortical atrophy. While multiple pathological factors may affect cortical atrophy, how and in which sequence potential cortical MR markers for cognitive decline are related to each other remains underexplored. Previous efforts to identify a sequence of MR markers related to the progression of cognitive and motor disability revealed early involvement of GM atrophy and structural disconnection in specific subcortical structures, such as the thalamus.^15^, ^16^ In contrast, the microstructural integrity of major WM tracts appeared to be affected relatively late, despite the early onset of WM lesions.^15^ While longitudinal study designs would be ideal to explore these concepts, advanced statistical methods applied to cross‐sectional data could provide first insights into a potential sequence of events, disentangling the clinical impact of neuroinflammatory and neurodegenerative changes. A better understanding of these inter‐relationships is key to eventually develop therapeutic targets and tools for disease monitoring.

Recent work has also shown the added value of clinically relevant regional atrophy measures, co‐occurring along major cortical networks in MS.^17^, ^18^ In fact, cognitive impairment has been related to alterations in specific cortical networks relevant for maintaining cognitive functioning, that is, the sensorimotor (SMN), default mode (DMN), and ventral attention network (VAN).^19^, ^20^ So, in MS, network‐specific explorations of cortical atrophy could provide insights into the identification of substrates of these network changes, underlying cognitive decline.^19^, ^20^ Together with the inconsistencies in the effect of CLs on cortical atrophy and their suggested (partly) independent role in cognitive decline, the interplay between CLs and cortical atrophy as MR markers for cognition may vary across networks.

Therefore, our aim was to assess the relationship between CLs and cortical thinning and volume loss in people with MS within cortical networks in lesional and normal‐appearing cortex, and second, how this relates to cognition at 5‐year follow‐up. We hypothesized that atrophy acts as a factor *in* the causal chain, that is, as “intermediate factor,” between CLs and cognition, especially within major cognitive networks. As such, we performed mediation analyses to assess the specific relationships between CLs, volume loss, and future cognition.

## Methods

### Participants

This observational longitudinal cohort study was approved by the institutional ethics review board. All participants provided written informed consent. Included participants were part of the Amsterdam MS cohort.^19^, ^21^ All data from this cohort have been used in previous publications studying MR volume measures in MS, but lesional and nonlesional cortical thinning and volume loss has not been investigated before. Participants were included if MR data were available at baseline and neuropsychological assessment data both at baseline and at 5‐year follow‐up. This resulted in the inclusion of 233 people with clinically definite MS^22^ and 60 healthy controls (HC). Participants with MS had no relapses or steroid treatment for at least 2 months prior to participation in both baseline and follow‐up visits. Exclusion criteria for all participants were the presence of other neurologic or neuropsychiatric disorder, and contraindication to MRI.

### Clinical and cognitive assessments

Clinical assessments were performed at baseline and 5‐year follow‐up. The Expanded Disability Status Scale (EDSS)^23^ was conducted by an experienced physician. A detailed description of the evaluation of cognitive functioning is reported in the Methods Supplement. Briefly, all participants underwent the expanded Brief Repeatable Battery of Neuropsychological tests at both time points.^24^ Tests were classified into seven predefined cognitive domains: attention, information processing speed, working memory, visuospatial memory, verbal memory, executive functioning (EF)—cognitive flexibility and verbal fluency, and EF—inhibition. Domain scores were corrected for the effects of age, sex, and education observed in normative data for the Dutch general population. An average cognition *Z*‐score was calculated as mean of the *Z*‐scores of individual cognitive domains. People with MS were classified as cognitively preserved (CP), mildly cognitively impaired (MCI), or cognitively impaired (CI) based on the presence of deviation in *Z*‐score relative to the performance of the HCs at the same time point (at *Z* ≤−1.5 for MCI, or *Z* ≤−2 for CI) on at least two out of seven domains.

### Image acquisition

All participants underwent 3 T MRI (GE Signa HDxt) at baseline, using an 8‐channel phased‐array head coil. The protocol included a 3D T1‐weighted fast spoiled gradient‐echo sequence for atlas segmentation and volume measures [repetition time/echo time/inversion time 7.8/3.0/450 ms, flip angle 12°, 0.94 × 0.94 × 1.0 mm^3^ voxel size], a 3D fluid‐attenuated inversion‐recovery (FLAIR) sequence for WM lesion segmentation (repetition time/echo time/inversion time 8000/125/2350 ms, 0.98 × 0.98 × 1.2 mm^3^ voxel size). For each MS participant, an artificially generated double inversion‐recovery (aiDIR) image was generated by a fully convolutional neural network for CL detection with the use of the 3D T1‐weighted and FLAIR sequences as inputs,^25^ which has recently been validated in a multicenter study.^26^

### Data processing

#### White and gray matter segmentation

In people with MS, FLAIR images were used to segment WM lesions by means of k‐nearest‐neighbor approach with tissue type priors.^27^ T1‐weighted images were filled using the WM lesion maps for maximizing further T1 processing steps.^28^ Cortical surface reconstruction was carried out by means of FreeSurfer (v7.0) with the T1‐weighted images as input. To ensure the accuracy of the cortical segmentation in the light of the presence of focal WM and cortical lesions, segmentations were checked for irregularities, corrected and rerun if errors occurred. After cortical surface reconstruction, the cortical GM surface was parcellated into 210 cortical GM regions (105 in each hemisphere) based on the Brainnetome atlas.^29^ This cortical surface atlas was resampled into a 3D volume and transformed into T1 space with nearest‐neighbor interpolation. Subcortical and deep GM regions were segmented in T1‐space with FIRST (v6.0), of which the bilateral hippocampi (as part of the DMN) were added to the GM atlas. This yielded a GM atlas of each participant consisting of 212 regions. All regions were then spatially assigned to one of the seven networks of functionally related regions based on the Yeo atlas^30^ for subsequent spatial analysis based on their spatial overlap: the DMN, dorsal attention (DAN), frontoparietal (FPN), limbic, SMN, VAN, and visual networks. The allocation of regions into networks is shown in Table S1.

#### Cortical lesion segmentation

CLs were scored and segmented on the aiDIR images with the use of Slicer^31^ (v5.2.1) by an experienced reader according to the MAGNIMS guidelines.^32^ Visual examples of CLs identified on the aiDIR images are displayed in Figure S1. aiDIR images were rigidly registered to their corresponding T1‐weighted image, after which the converted matrix was applied to the GM atlas, using nearest‐neighbor interpolation, to register the atlas to aiDIR space. Presence and volume of CLs within all 212 cortical regions were assessed by combining the CL mask and atlas. For the seven networks, lesional and normal‐appearing cortical regions within the individual networks were identified based on the presence of CLs in the 212 cortical regions.

#### Cortical volume measures

Cortical thickness and volume are two widely used measures for analyzing cortical structural morphometry in neuroimaging research. Previous neuroimaging work demonstrates that these phenotypically independent measures should be considered separately.^33^, ^34^ To explore and compare potential differences in clinical relevance, both cortical thickness and volume were evaluated in subsequent analyses. Cortical volume was calculated in all 212 regions, normalized by the intracranial volume provided by FreeSurfer, as recommended by previous literature.^34^, ^35^ The hippocampi are included for volumetric analyses as GM structures, but are not included by FreeSurfer due to the complex morphology. Hence, cortical thickness could only be calculated in the parahippocampal gyrus and not in the hippocampus itself. In network analyses, the DMN therefore only included hippocampus data considering the normalized cortical volumes.

For people with MS, values were transformed to a *Z*‐score based on the regional mean and standard deviation of the HC to adjust for the physiological regional variation in cortical thickness and volume. Normalized volume and thickness *Z*‐scores values were averaged across regions within cortical networks and across the whole brain. Additionally, mean *Z*‐scores were calculated for lesional and normal‐appearing cortical regions within each individual network and at whole‐brain level separately.

### Statistical analysis

R (v4.2.1) was used for statistical analyses. To test normality, we used Kolmogorov–Smirnov testing and histogram inspection of the variable distribution. Due to right‐skewed data, CL volumes were log(× + 1) transformed and WM lesion volume log(×) transformed for subsequent analysis. We analyzed baseline characteristics with descriptive statistics. Demographics and imaging features were expressed as *N* (%), mean ± SD, or Median (IQR), depending on normality and type. Level of education was dichotomized and defined as high educational level corresponding to ≥6 on the Verhage scale.^36^ Of all analyses, effect sizes (95% confidence interval) are reported. *P*‐values are Bonferroni‐corrected for multiple comparisons with significance level 0.05, reported in the results section as *P*_corr_.

#### Relationship between volume changes and cortical lesions within the same individual

We performed multivariable linear regression models with the log‐transformed CL volume as independent variable explaining either cortical volume or thickness. The *Z*‐scores of mean cortical thickness and volume were included as dependent variables in two separate models. Age and sex were included as covariates. Similar regression models were performed within cognitive subgroups (CP, MCI, and CI).

Then, we compared the regional cortical volume and thickness between normal‐appearing and lesional cortical regions by applying paired *t*‐tests. Additionally, to test whether found relationships between CL volume and cortical volume and thickness vary across functional brain networks, paired *t*‐tests were repeated within each functional network separately. Due to the within‐subject nature of these analyses, people with MS without CLs were excluded from this part of the analysis (*N* = 31).

#### Global volume changes and cognitive status at baseline

Next, the mean *Z*‐scores of cortical volume and thickness were further analyzed between cognitive subgroups using multinominal logistic regression models, adjusting for age, sex, and high level of education. As these demographics may impact both cortical volume metrics and cognitive functioning,^37^, ^38^, ^39^ these variables were considered potential confounders in our models with cognitive outcomes. Significant results were further explored in multivariable logistic regression models to test whether changes in volume or thickness between cognitive subgroups were driven by lesional and/or normal‐appearing cortex. Volume or thickness of both lesional and normal‐appearing cortical regions, including the covariates age, sex, and high level of education, were included as independent variables in the model.

#### Global volume changes and cognitive status at 5‐year follow‐up

We then aimed to identify MR predictors among volume and thickness measures at baseline for cognitive functioning at follow‐up. Similar logistic regression models were performed with the use of the classification of cognitive subgroups at 5‐year follow‐up as dependent variable. Models were adjusted for age, sex, and high level of education and cognitive subgroups at baseline. The latter was included to limit bias related to the probability of belonging to a particular subgroup based on the initial (baseline) classification.

#### Network volume changes and cognitive status at 5‐year follow‐up

The measure of lesional and/or normal‐appearing cortex showing the strongest association with the cognitive classification at 5‐year follow‐up was spatially assessed across functional networks using the same statistical approach. This step was performed in order to assess network‐specific preferences of neurodegeneration relating to future cognitive decline.

#### Network volume changes and cognitive functioning at 5‐year follow‐up

To determine the network measure most relevant for explaining cognition, a linear regression model with forward stepwise selection approach (*α*‐entry = 0.05) was performed, including the covariates age, sex, high level of education, and baseline cognitive functioning. Network measures added to this regression model include the network measures of lesional and/or normal‐appearing cortex showing the strongest association with the cognitive status at 5‐year follow‐up (as described in section “Network volume changes and cognitive status at 5‐year follow‐up”). This approach was chosen to limit potential suspected multicollinearity between volume or thickness measures. To evaluate any preferential susceptibility to malfunctioning of a particular cognitive domain by volume loss of a specific subnetwork, this approach was repeated for all seven cognitive domains.

To assess whether the found relationships between network volume changes and cognitive functioning at follow‐up are independent of WM lesions, the final regression models were repeated with the inclusion of WM lesion volume as covariate.

#### Mediation analysis

**Figure 1:**
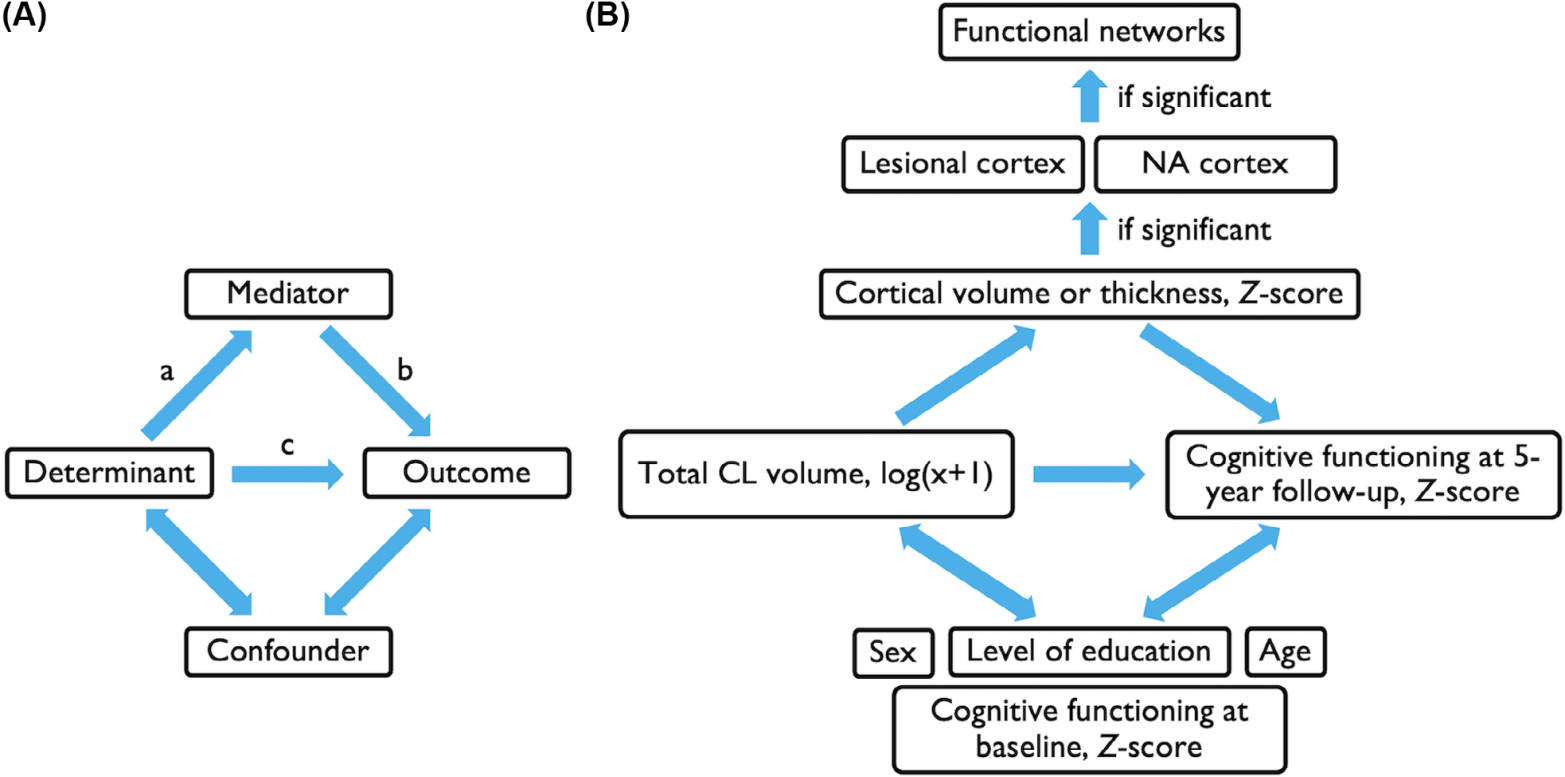
Schematic overview of mediation analysis. (A) Displays the schematic concept of a mediation analysis: a = the path from the determinant to the mediator, b = the path from the mediator to the outcome, and c = the *direct* path (i.e., direct effect) from the determinant to the outcome variable. The *indirect* path (i.e., indirect effect) in a mediation analysis consists of the paths a and b. The *total* effect in a mediation analysis is c + (a × b). (B) Displays the mediation analyses including confounders performed in present study. NA, normal‐appearing.

Finally, we tested the hypothesis that the relationship between CL volume and cognitive decline is *mediated* by cortical volume loss. Structural Equation Modeling was applied with the “sem” function of Lavaan^40^ (Fig. 1A). In our analysis, we used the log‐transformed CL volume as determinant, *Z*‐transformed volume loss as mediator, and cognition as outcome variable (Fig. 1B). Mediation analyses were performed for cognitive functioning at baseline and at 5‐year follow‐up to assess how CL volume might influence cognitive functioning to a variable degree over time. All mediation analyses were adjusted for age, sex, and level of education. Baseline cognitive functioning was included in the models for follow‐up cognition to ensure we were assessing future cognitive performance rather than merely reflecting baseline performance.

For the significant total effects that are mediated by an indirect effect (*P* < 0.05), mediation analyses were repeated including *Z*‐score of both lesional and normal‐appearing cortical volume as separate mediator variables. Using this approach, two indirect effects instead of one were tested.

To assess whether the found indirect effects via cortical volume is independent of WM lesions, mediation analyses were again repeated with the inclusion of WM lesion volume as covariate.

Finally, we targeted the analysis based on the found associations between cortical volume and cognitive status (as described in section “Network volume changes and cognitive status at 5‐year follow‐up”). Mediation analyses were repeated with the inclusion of the candidate cortical network MR markers as individual indirect effects to detect any preferential paths (i.e., cortical networks) through which CLs affect cognitive functioning of specific domains.

## Results

### Demographics

Three out of 233 people with MS (1.3%) were excluded from subsequent analyses due to missing neuropsychological assessment data of five or more domains.

Of the remaining 230 people with MS at baseline, 179 (77.8%) had relapsing–remitting MS, and 18 (7.8%) primary‐progressive and 33 (14.3%) secondary‐progressive MS (Table 1). At baseline, people with MS had a mean age of 47.6 ± 10.9 years old, mean disease duration of 11.7 ± 6.6 years, and median EDSS of 3.0 [interquartile range (IQR) 2.0]. Normalized GM volumes (MS: 0.42 ± 0.03 and HC: 0.43 ± 0.02, *P* = 0.002), especially deep GM volumes (MS: 0.034 ± 0.003 and HC: 0.037 ± 0.002, *P* = 5.67⋅10^−17^), were significantly lower in MS compared to HC. Of clinical characteristics, age differed significantly between cognitive groups, though without post hoc differences between pairs of subgroups. CI‐MS had higher EDSS scores compared to CP‐MS. Cognitive groups also differed in WM lesion volume and normalized brain volumes, with highest lesion and lowest brain volumes found in CI‐MS. *Z*‐scores per cognitive domain at both time points are reported in Table S2.

**Table 1:** Demographics of people with multiple sclerosis and healthy volunteers.

	HC	MS	HC versus MS	CP‐MS	MCI‐MS	CI‐MS	Group differences
			Test statistic, *P*‐value				Test statistic, *P*‐value
*N* at baseline (%)	60	230		123 (53.5)	58 (25.2)	49 (21.3)	
*N* at FU (%)	60	230		124 (53.9)	45 (19.6)	61 (26.5)	
Time interval between baseline and FU (years)	5.45 (1.08)	4.80 (0.83)		4.86 (0.85)	4.75 (0.80)	4.71 (0.82)	
*Demographics*							
Age at baseline (years)	46.5 (9.9)	47.6 (10.9)	*t* (288) = −0.75, *P* = 0.460	46.8 (9.6)	46.3 (12.1)	51.2 (12.1)	*F* (2,227) = 3.41, *P* = **0.035**
Sex (female), *N* (%)	31 (51.7)	155 (67.4)	*χ* ^2^ (1)=5.12, *P* = **0.024**	88 (71.5)	38 (65.5)	29 (59.2)	*χ* ^2^ (2)=2.56, *P* = 0.278
Level of education (high^a^), *N* (%)	36 (60.0)	101 (43.9)	*χ* ^2^ (1)=4.94, *P* = **0.026**	58 (47.2)	24 (41.4)	19 (38.8)	*χ* ^2^ (2)=1.20, *P* = 0.549
*MS characteristics*							
MS subtype at baseline (RR/SP/PP), *N* (%)	‐	179 (77.8)/33 (14.3)/18 (7.8)	‐	101 (82.1)/15 (12.2)/7 (5.7)	44 (75.9)/9 (15.5)/5 (8.6)	34 (69.4)/9 (18.4)/6 (12.2)	*χ* ^2^ (4)=3.76, *P* = 0.440
MS subtype at FU (RR/SP/PP), *N* (%)	‐	158 (68.7)/54 (23.5)/18 (7.8)	‐	94 (75.8)/25 (20.2)/5 (4.0)	31 (68.9)/9 (20.0)/5 (11.1)	33 (54.1)/20 (32.8)/8 (13.1)	*χ* ^2^ (4)=10.95, *P* = **0.027**
EDSS at baseline^b^	‐	3.0 [2.0]	‐	3.0 [2.0]	3.25 [1.5]	3.5 [2.5]	*H* (2)=8.38, *P* = **0.015** ^d^
EDSS at FU^b^		3.5 [2.5]		3.0 [1.5]	4.0 [2.0]	4.0 [3.0]	*H* (2)=12.82, *P* = **0.002** ^d^
Disease duration at baseline (years)	‐	11.7 (6.6)	‐	11.5 (6.5)	10.7 (7.1)	13.4 (6.5)	*F* (2,220) = 2.28, *P* = 0.104
Medication at baseline (yes), *N* (%)	‐	85 (29.5)	‐	42 (34.1)	27 (46.6)	15 (30.6)	*χ* ^2^ (2)=3.55, *P* = 0.169
First‐line treatment		69 (24.0)		33 (26.8)	23 (39.7)	12 (24.5)	
Second‐line treatment		16 (5.6)		9 (7.3)	4 (6.9)	3 (6.1)	
*MRI characteristics*							
NGM volume	0.43 (0.02)	0.42 (0.03)	*t* (288) = 3.05, *P* = **0.002**	0.43 (0.02)	0.42 (0.02)	0.41 (0.03)	*F* (2,227) = 6.20, *P* = **0.002** ^**c**^
NDGM volume	0.037 (0.002)	0.034 (0.003)	*t* (148.4) = 9.48, *P* = **5.67⋅10** ^**−17**^	0.035 (0.003)	0.034 (0.003)	0.032 (0.004)	*F* (2,227) = 14.60, *P* = **1.00⋅10** ^**−6**^ ^d^, ^e^
NWM volume	0.30 (0.02)	0.28 (0.02)	*t* (128.3) = 6.29, *P* = **4.63⋅10** ^**−9**^	0.29 (0.02)	0.28 (0.02)	0.27 (0.02)	*F* (2,227) = 11.21, *P* = **2.30⋅10** ^**−5**^ ^d^, ^e^
WM lesion volume (mL)^b^, ^c^	‐	9.22 [13.89]	‐	8.65 [8.59]	10.36 [16.01]	13.46 [21.00]	*F* (2,227) = 5.06, *P* = **0.007** ^**d**^

Demographics of healthy volunteers (HC) and people with multiple sclerosis (MS), including cognitive subgroups: cognitively preserved (CP), mildly cognitively impaired (MCI) and cognitively impaired (CI) people with multiple sclerosis (MS). Values are Mean (SD) unless specific otherwise. Significant *P*‐values are marked in bold.

EDSS, expanded disability status scale; FU, follow‐up; NDGM, normalized deep gray matter volume; NGM, normalized total gray matter volume; NWM, normalized white matter; PP, primary progressive; RR, relapsing–remitting; SP, secondary progressive.

^a^ Level of education was dichotomized and defined as high educational level corresponding to ≥6 on the Dutch Verhage scale [Verhage F. Intelligence and Age in a Dutch Sample. 8 (4): 238–245].

^b^ Median [interquartile range].

^c^ Comparison analyses performed on log‐scale.

^d^ Significant post hoc difference (Bonferroni‐corrected) between CP‐ and CI‐MS.

^e^ Significant post hoc difference (Bonferroni‐corrected) between CP‐ and MCI‐MS.

At 5‐year follow‐up, 124 people with MS were classified as CP, 45 people as MCI, and 61 people as CI. Of the 123 CP‐MS at baseline, 100 people with MS (81.3%) were still CP at 5‐year follow‐up, 14 people (11.4%) converted to MCI, and nine (7.3%) to CI. The 58 MCI‐MS at baseline remained MCI at follow‐up in 21 cases (36.2%), whereas 19 people (32.8%) converted to CI‐MS; in 18 people (31.0%), cognitive functioning improved to the level of CP‐MS. As such, 33 of 49 CI‐MS at baseline (67.3%) remained CI at follow‐up, whereas in 16 people (32.6%) their cognitive performance improved to the level of CP (*N* = 6; 12.2%) or MCI (*N* = 10; 20.4%).

Regarding CLs, 199 people with MS had at least one CL with a median volume of 0.12 mL (IQR 0.26) at baseline. Of the 31 people with MS without CLs visible on 3 T MRI, 16 (51.6%) were CP‐MS, 11 (35.5%) MCI‐MS and four (12.9%) were CI‐MS. Comparisons between people with MS with and without CLs are reported in Table S3. People with CLs have longer disease duration and more progressive MS disease types, with lower normalized deep GM volumes and higher WM lesion volumes compared to those without.

**Figure 2:**
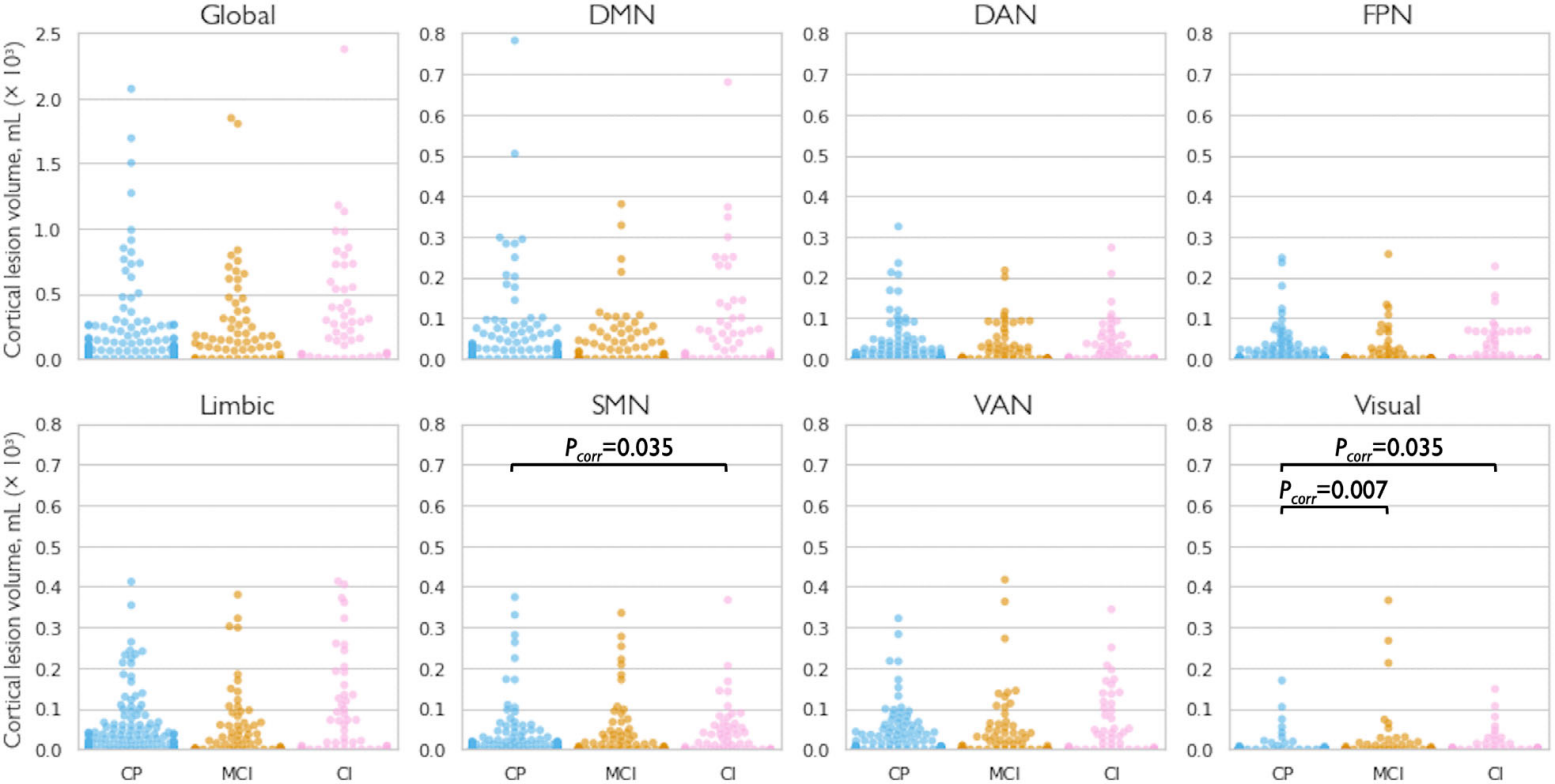
Distribution of cortical lesions across cortical networks. Swarm plots of cortical lesion volumes in the entire cortex (referred to as “global”) and in each individual cortical network in people with multiple sclerosis, divided in three cognitive subgroups: cognitively preserved (CP), mildly cognitively impaired (MCI), and cognitively impaired (CI) patients. Brackets with adjusted *P*‐values denote significant differences in log‐transformed cortical lesion volume between groups. Detailed statistics are reported in Table S4. DAN, dorsal attention network; DMN, default mode network; FPN, frontoparietal network; SMN, sensorimotor network; VAN, ventral attention network.

Prevalence of CLs was highest in the limbic network (73.0% of people with MS), followed by the DMN (70.9%; Fig. 2, Table S4). CL volumes relative to network volume were highest in the limbic network (1.17⋅10^−3^ ± 1.72⋅10^−3^) and VAN (9.98⋅10^−4^ ± 1.72⋅10^−3^) compared to other networks (DAN: 5.45⋅10^−4^ ± 9.75⋅10^−4^, DMN: 5.32⋅10^−4^ ± 9.47⋅10^−4^, FPN: 3.96⋅10^−4^ ± 8.09⋅10^−4^, SMN: 5.62⋅10^−4^ ± 1.05⋅10^−3^), and lowest in the visual network (1.50⋅10^−4^ ± 5.51⋅10^−4^). Between cognitive subgroups, CL volume was higher in CI‐MS than CP‐MS in the SMN [CP‐MS: 3.0(IQR 23.0) and CI‐MS: 27.0(IQR 61.0), *P*_corr_ = 0.035] and visual network [CP‐MS: 0.0(IQR 0.0) and CI‐MS: 0.0(IQR 14.0), *P*_corr_ = 0.035]. MCI‐MS [0.0(IQR 12.0)] had higher CL volumes only in the visual network than CP‐MS (*P*_corr_ = 0.007).

### Cortical thickness and volume in lesional cortex

**Figure 3:**
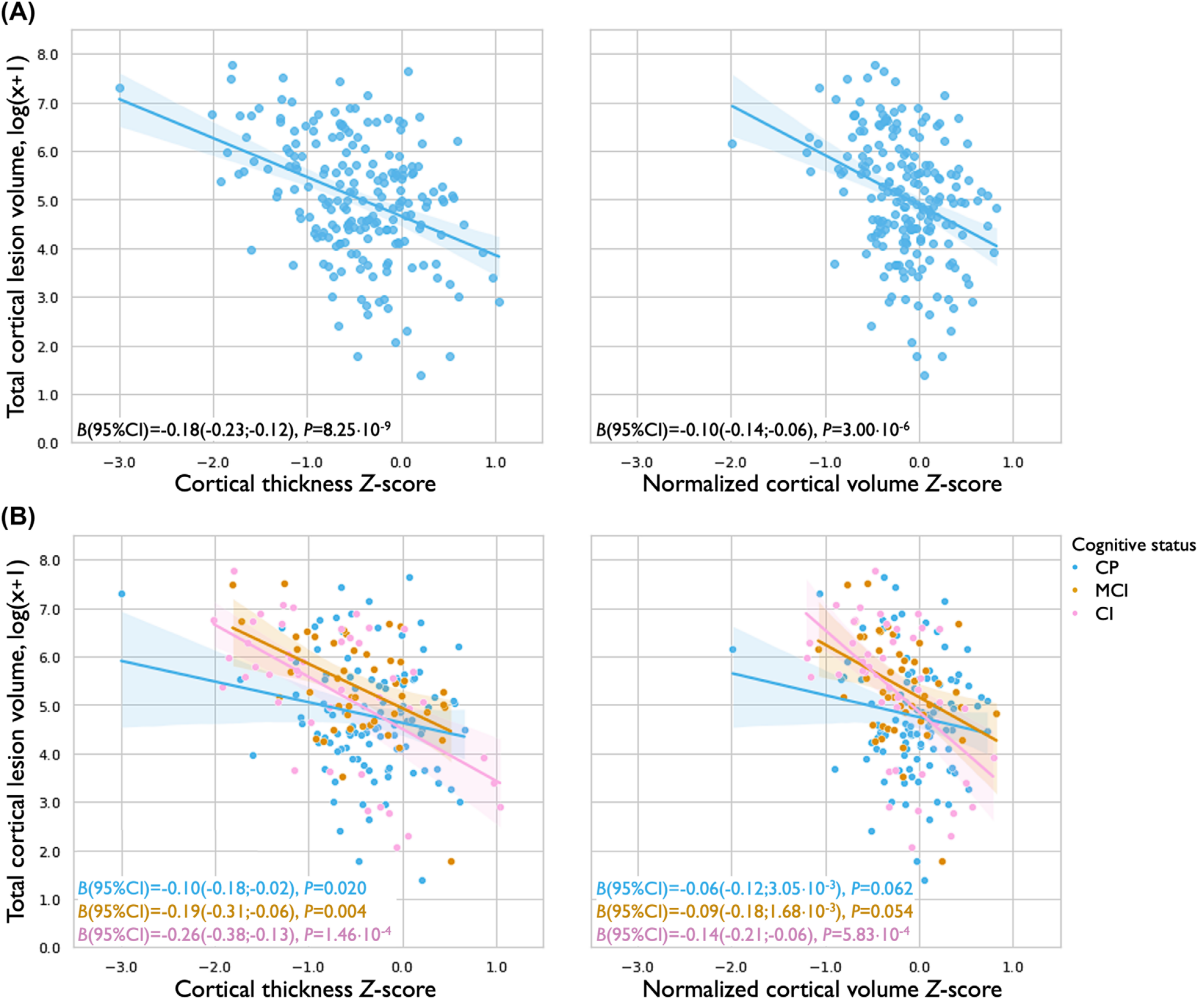
Relationship between cortical lesion volume and cortical measures. Whole‐brain cortical thickness *Z*‐score (left) and whole‐brain normalized cortical volume (right) are shown on the *x*‐axis, log‐transformed total cortical lesion volume on the *y*‐axis. Regression plots are displayed for the entire sample of people with multiple sclerosis (A) and for each cognitive subgroup separately (B). Unstandardized *B* with corresponding 95% confidence intervals (CI) and unadjusted *P*‐values are displayed in both subplots. people with multiple sclerosis without cortical lesions were excluded from the regression analyses, and therefore not shown in the figure.

Multivariable linear regression, adjusting for age and sex, showed a negative association between total CL volume and whole‐brain cortical thickness *Z*‐score [*B* (95% confidence interval) = −0.18 (−0.23 to −0.12), *P*_corr_ = 1.65 10^−8^] and cortical volume *Z*‐score [*B* (95% confidence interval) = −0.10 (−0.14 to −0.06), *P*_corr_ = 6.00 10^−6^; Fig. 3A]. These associations were most pronounced in CI‐MS [cortical thickness: *B* (95% confidence interval) = −0.26 (−0.38 to −0.13), *P*_corr_ = 0.001; normalized cortical volume: *B* (95% confidence interval) = −0.14 (−0.21 to −0.06), *P*_corr_ = 0.003], followed by MCI‐MS [cortical thickness: *B* (95% confidence interval) = −0.19 (−0.31 to −0.06), *P*_corr_ = 0.024; normalized cortical volume: *B* (95% confidence interval) = −0.09 (−0.18 to 1.68 10^−3^), *P*_corr_ = 0.325] and CP‐MS [cortical thickness: *B* (95% confidence interval) = −0.10(−0.18 to −0.02), *P*_corr_ = 0.122; normalized cortical volume: *B* (95% confidence interval) = −0.06(−0.12 to 3.05⋅10^−3^), *P*_corr_ = 0.374; Fig. 3B].

Cortical thickness *Z*‐score was significantly lower in lesional compared to normal‐appearing cortex [lesional: −0.70 ± 0.84 and normal‐appearing: −0.45 ± 0.60, *P*_corr_ = 2.36⋅10^−10^]. Across networks, cortical thickness was significantly lower in lesional compared to normal‐appearing cortex in all networks [DAN—lesional: −0.91 ± 1.21 and normal‐appearing: −0.65 ± 0.75, *P*_corr_ = 0.002; DMN—lesional: −0.66 ± 0.91 and normal‐appearing: −0.50 ± 0.63, *P*_corr_ = 0.018; FPN—lesional: −0.80 ± 1.20 and normal‐appearing: −0.55 ± 0.73, *P*_corr_ = 0.016; limbic—lesional: −0.83 ± 1.07 and normal‐appearing: −0.52 ± 0.67, *P*_corr_ = 1.40⋅10^−5^; VAN—lesional: −0.63 ± 1.01 and normal‐appearing: −0.42 ± 0.63, *P*_corr_ = 0.016; visual—lesional: −0.89 ± 1.17 and normal‐appearing: −0.55 ± 0.71, *P*_corr_ = 0.023], except for a trend for the SMN [lesional: −0.78 ± 1.20 and normal‐appearing: −0.65 ± 0.78, *P* = 0.059]. Cortical volumes of lesional cortex were similar to volumes of normal‐appearing cortex [lesional: −0.10 ± 0.71 and normal‐appearing: −0.15 ± 0.41, *P* = 0.178].

### Cognition‐related cortical thickness and volume at baseline

At baseline, whole‐brain cortical thickness *Z*‐scores relative to HC showed a significant difference between CP‐MS and CI‐MS [OR (95% confidence interval) = 0.35 (0.18–0.67), *P*_corr_ = 0.003; Table S5]. Subanalyses of normal‐appearing and lesional cortex were performed by including both variables as independent variables in logistic regression models. The significant difference in cortical thickness between CP‐MS and CI‐MS seemed driven by thinning of the normal‐appearing cortex in CI‐MS [OR (95% confidence interval) = 0.31 (0.10–0.93), *P* = 0.036] instead of lesional cortex [OR (95% confidence interval) = 1.22 (0.58–2.59), *P* = 0.598]. The *P*‐values for the comparisons of *Z*‐scores of normalized cortical volume did not reach significance between any of the cognitive subgroups after applying the Bonferroni correction.

### Cognition‐related cortical thickness and volume at follow‐up

**Figure 4:**
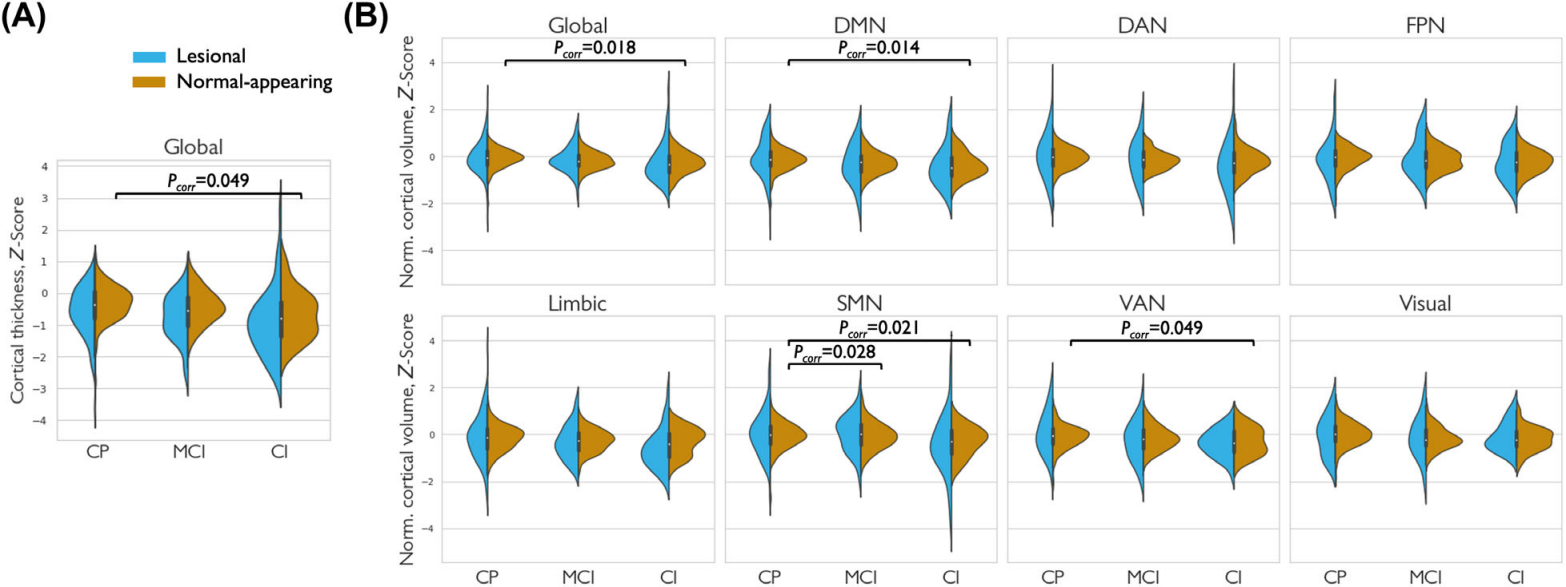
Baseline cortical thickness and volume among cognitive subgroups at 5‐year follow‐up. Violin plots of the mean cortical thickness (A) of the entire cortex (referred to as “global”) and the normalized cortical volume (B) of the entire cortex and in each individual cortical network in people with multiple sclerosis, divided in three cognitive subgroups: cognitively preserved (CP), mildly cognitively impaired (MCI), and cognitively impaired (CI) patients. Violin plots are split based on the division of cortical regions with cortical lesions (referred to as “lesional”) and without cortical lesions (“normal‐appearing”). Brackets with adjusted *P*‐values denote significant differences in log‐transformed cortical lesion volume between groups. Detailed statistics are reported in Table S6. DAN, dorsal attention network; DMN, default mode network; FPN, frontoparietal network; SMN, sensorimotor network; VAN, ventral attention network.

People with CI‐MS at 5‐year follow‐up showed significantly lower baseline whole‐brain cortical thickness and smaller baseline cortical volume *Z*‐scores relative to HC compared to CP‐MS [OR (95% confidence interval) = 0.35 (0.16–0.76), *P*_corr_ = 0.015, and OR (95% confidence interval) = 0.08 (0.02–0.31), *P*_corr_ = 3.62 10^−4^, respectively]. Similarly, people with MCI‐MS showed lower whole‐brain cortical volume *Z*‐scores compared to CP‐MS [OR (95% confidence interval) = 0.16 (0.05–0.56), *P*_corr_ = 0.008; Table S6]. Next, we evaluated thickness and volumetric effects in lesional and normal‐appearing cortex separately. Only the normal‐appearing cortex had a significant effect on the distinction between CP‐MS and CI‐MS for both cortical thickness [OR (95% confidence interval) = 0.21 (0.06–0.82), *P*_corr_ = 0.049] and volume [OR (95% confidence interval) = 0.10 (0.02–0.57), *P*_corr_ = 0.018; Fig. 4, Table S6].

As the normalized cortical volume showed a stronger association (in terms of odds ratio) with cognitive subgroups at follow‐up compared to cortical thickness while both were significant, we subsequently only assessed changes in normalized cortical volume *Z*‐scores at the network‐level. In all networks, except the FPN, cortical network volume was significantly lower in CI‐MS compared to CP‐MS (for detailed statistics, see Table S6). MCI‐MS had lower cortical network volume of the SMN, VAN, and limbic network compared to CP‐MS. Focusing on the distinction between lesional and normal‐appearing cortex, only volume loss of the normal‐appearing cortex within the DMN, DAN, limbic, SMN, and VAN networks was significantly associated with cognitive status, instead of volume loss of lesional cortex. After Bonferroni correction, network volume loss of normal‐appearing cortex in CP‐MS compared to CI‐MS remained significant in the DMN, SMN, and VAN [OR (95% confidence interval) = 0.13 (0.03–0.46), *P*_corr_ = 0.014, OR (95% confidence interval) = 0.14 (0.04–0.51), *P*_corr_ = 0.021, and OR (95% confidence interval) = 0.16(0.04–0.61), *P*_corr_ = 0.049, respectively; Fig. 4].

**Figure 5:**
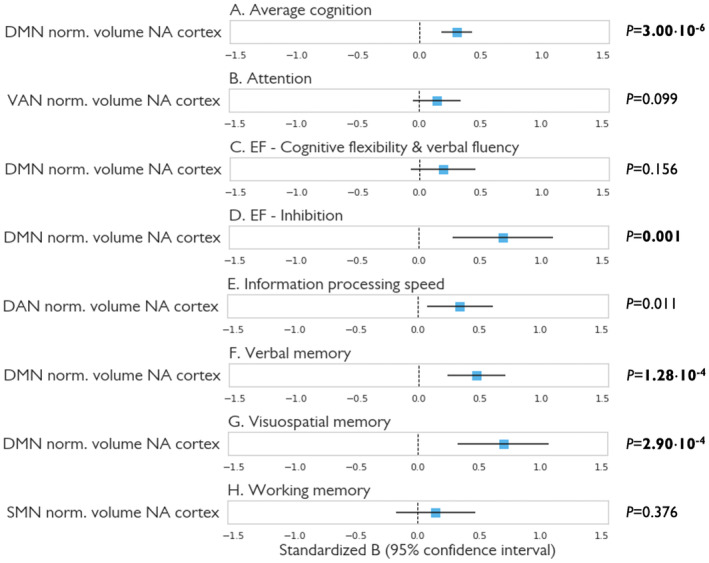
Prediction of cortical normalized volumes for cognitive functioning at 5‐year follow‐up. Results of the linear regression models based on forward selection. Age, sex, level of education, and baseline cognitive functioning were entered in a single step in the first block (results not reported), after which the significant volumetric MR variables [normalized volumes of normal‐appearing (NA) cortex of default mode (DMN), dorsal attention (DAN), limbic, sensorimotor, and ventral attention (VAN) networks] were entered by means of a forward stepwise selection approach. Unstandardized *B* with corresponding 95% confidence intervals and *P*‐values of the MR variables included in the final model are displayed in subplots, per cognitive domain. Significant *P*‐values surviving Bonferroni correction for multiple testing (*P* < 6.25⋅10^−3^) are marked in bold. EF, executive functioning.

Finally, we investigated which combination of candidate network volume measures (normal‐appearing cortex of the DMN, DAN, limbic, SMN, and VAN) best predicted future cognition using forward stepwise linear regression (Fig. 5). Average cognition at 5‐year follow‐up was best predicted by baseline normalized network volume of normal‐appearing cortex of the DMN [*B* (95% confidence interval) = 0.31 (0.18–0.43), *P*_corr_ = 2.40⋅10^−5^]. After adding WM lesion volume to the model, worse average cognition at 5‐year follow‐up could be independently explained by both lower baseline normalized network volume of normal‐appearing cortex of the DMN [*B* (95% confidence interval) = 0.24 (0.11–0.37), *P* = 0.001] and higher WM lesion volume [*B* (95% confidence interval) = −0.21 (−0.35 to −0.06), *P* = 0.006].

Focusing on individual cognitive domains, volume loss of the normal‐appearing cortex of the DMN was significantly related to future cognitive functioning in the domains verbal memory [*B* (95% confidence interval) = 0.47 (0.23–0.71), *P*_corr_ = 0.001], visuospatial memory [*B* (95% confidence interval) = 0.70 (0.32–1.07), *P*_corr_ = 0.002], and EF—inhibition [*B* (95% confidence interval) = 0.69 (0.27–1.10), *P*_corr_ = 0.010]. Normal‐appearing cortex of the DAN tended to be associated with information processing speed, but did not survive Bonferroni correction [*B* (95% confidence interval) = 0.35 (0.08–0.61), *P*_corr_ = 0.088].

### Mediation analysis

At baseline, CL volume was associated with cognitive functioning, reflected by a significant total effect on average cognition (*P* = 1.22*⋅*10^−4^). The total effect of CL volume on average cognition was explained by a significant direct effect of CLs themselves (*P* = 0.001) and a trend toward an indirect effect through normalized cortical volume (*P* = 0.089).

Next, to identify a potential sequence of events for future cognitive functioning, we tested the hypothesis that the relationship between CLs and cognitive functioning at follow‐up is mediated by cortical volume loss. To narrow these subsequent analyses, cognitive domains included here are all domains showing associations (*P*_corr_ < 0.10) with cortical volume loss in the linear regression models above. To evaluate the impact of CL volume on specifically *future* cognitive performance, baseline cognitive functioning was included as a covariate in the mediation analyses. Results with detailed statistics are reported in Table S7.

Based on the mediation analyses, CL volume was related to cognitive functioning at 5‐year follow‐up, reflected by the significant total effect with average cognition (*P*_corr_ = 0.015). Specifically, CL volume showed associations with cognitive decline in information processing speed (*P*_corr_ = 0.049) and verbal memory (*P*_corr_ = 0.035) and a trend toward an association with decline in visuospatial memory (*P*_corr_ = 0.080). In contrast to the effects of CLs on cognition at baseline, the total effect of CL volume on average cognition was explained by cortical volume loss, as the indirect effect showed significance (*P*_corr_ = 4.99*⋅*10^−4^) whereas the direct effect did not (*P* = 0.293). Similar effects were seen for specifically verbal and visuospatial memory (both *P*_corr_ = 0.005), though, for visuospatial memory, without a significant total effect which weakens the interpretation of the indirect effects. We then included the normalized cortical volume of normal‐appearing as well as lesional cortex as separate mediators in the mediation analyses showing an indirect effect of CL volume on cognition *via* normalized cortical volume (for detailed statistics, see Table S8). Results show a significant association between CL volume and average cognition through an indirect effect of normal‐appearing cortex volume (*P*_corr_ = 0.001) as opposed to an indirect effect of lesional cortex volume (*P* = 0.416) or a direct effect of CL volume (*P* = 0.315). So, the observed indirect effects were driven by an indirect effect specifically of normal‐appearing cortex volume.

We then aimed to assess whether the found indirect effects of cortical volume loss were independent of WM lesion burden. The effects of CL volume on average cognition (*P*_corr_ = 0.004), and information processing speed (*P*_corr_ = 0.008), verbal (*P*_corr_ = 0.008), and visuospatial memory (*P*_corr_ = 0.032) were still explained by indirect effects of normal‐appearing cortex volume (Table S9).

Finally, mediation analyses were performed for volume loss within relevant cortical networks (i.e., the DMN, DAN, limbic, SMN, and VAN) that predicted 5‐year cognitive performance. Cognitive domains that could be predicted by any of the network volumes (i.e., average cognition, and individual domains EF—inhibition, information processing speed, visuospatial memory and verbal memory) were included as outcome variables in five mediation analyses. Indirect effects of CL volume on *average cognition* via normal‐appearing cortex volume found at whole‐brain level only remained significant for normal‐appearing cortex volume of the SMN (*P*_corr_ = 0.020; Table S10). Similar results were found for *verbal* (*P*_corr_ = 0.049) and *visuospatial memory (P*_corr_ = 0.040), though the total effect of CL volume on visuospatial memory did not reach significance.

## Discussion

In this work, we assessed how cortical thinning and volume loss in people with MS is related to the presence of CLs across distributed cortical networks, and whether the relationship between CLs and cognition is mediated by volume loss. In our cohort, CL volume was associated with cortical thickness and volume. Whereas cortical *thinning* was more pronounced in lesional compared to normal‐appearing cortex, cortical *volume loss* of particularly normal‐appearing cortex was more strongly associated with future cognition than cortical thinning. Distinctly, volume loss of normal‐appearing cortex of the DMN, SMN, and VAN was related to cognitive impairment. Volume loss of the DMN seemed particularly relevant for inhibition, and verbal and visuospatial memory. Based on mediation analyses, the effect of CL volume on future cognition could be explained by an indirect effect through cortical volume loss, instead of by a direct effect of CL volume. Remarkably, this mediating effect was independent from WM lesion volume.

CL volume was associated with both cortical thinning and volume loss. Cortical thinning was more pronounced in lesional compared to normal‐appearing cortex, except for regions related to the SMN. The relationship between CL volume and cortical atrophy is inconsistent in the literature with CLs being either independent from or negatively associated with cortical atrophy.^10^, ^11^, ^13^, ^14^ Based on previous cross‐sectional 7 T and histopathology studies, the spatial occurrence of cortical neurodegeneration seems to not directly relate to CL density, suggestive for a reduced role of primary cortical demyelination in cortical neurodegeneration.^9^, ^14^ However, as 7 T MRI is known for its high sensitivity to subpial lesions,^41^ the lack of an association between CLs and atrophy at 7 T MRI found previously might be (at least partially) driven by subpial lesions. Therefore, CL subtypes could possibly affect cortical neurodegeneration to a different degree, warranting future studies focusing on the differential impact of CL subtypes on cortical neurodegeneration. Inexplicably, the SMN was the only network without significant cortical thinning of lesional cortex, although the effect did show a trend. The supplementary motor cortex and precentral gyrus are identified as regions consisting of the highest CL burden on 7 T MRI,^42^ which is not seen in our sample. This discrepancy might have biased our classification of lesional and normal‐appearing cortex; with the latter consisting of a large number of undetected CLs. Additionally, our analyses solely focused on the relationship between CLs and cortical neurodegeneration, but cortical pathology alone cannot explain the full extent of cortical neurodegeneration. More severe cortical thinning may also be a consequence of a higher WM lesion load in WM tracts connected to specific cortices, potentially leading to retrograde neurodegeneration.^43^ Future research combing WM tractography, lesion load, and cortical atrophy measures should explore the independent contributions of focal cortical demyelination and retrograde degeneration by WM disconnection to cortical atrophy.

Next, we found that cortical thickness and volume do not necessarily relate to cognitive impairment in the same way, which were previously indicated to be phenotypically independent of each other.^34^ Whereas both measures were related to CL volume, lesional cortex could be distinguished from normal‐appearing cortex by cortical thinning more so than cortical volume loss. As within‐subjects analyses evaluate data points within the same individual, these analyses could not have been biased by inter‐subject variations in intracranial volume. Cortical thinning can possibly better capture subtle changes of neurodegenerative alterations, compared to normalized volume. Interestingly, in our cohort, normalized cortical volume showed a larger effect in differences between cognitive groups at follow‐up compared to cortical thickness, of which differences were already evident at baseline. As subgroups shifted during follow‐up, differences in cognitive performance and MR characteristics might become more distinct as disease progresses. Detecting cognitive deterioration over time might require more substantial neurodegenerative alterations reflected by changes in cortical volume along with cortical thinning. From a clinical perspective, cortical volume would therefore be a more suitable measure to predict long‐term rather than baseline or short‐term cognition.

In our cohort, cortical volume alterations between people with and without cognitive impairment were driven by volume loss of normal‐appearing instead of lesional cortex. One might argue that the relevance of volume loss of normal‐appearing cortex might be due to undetected CLs on aiDIR; in other words, that the observed effects might still be caused by CLs themselves, although this would not explain the lack of a relationship within lesional cortex. In addition, based on histopathology, the neurodegenerative changes seen in subpial lesions and normal‐appearing cortex do not differ in neuron and axon density.^14^ Thus, undetected subpial lesions may not impact the interpretability of our results in normal‐appearing cortex. Of all networks, normal‐appearing cortex volume loss of the DMN best predicted cognitive functioning at follow‐up. From an anatomical perspective, the DMN consists of among others the superior temporal and posterior cingulate cortex, which show early atrophy in MS and have been related to cognitive performance.^18^, ^44^ It has even been suggested that disconnection in the DMN may deprive the compensatory mechanism that people with MS use to counteract widespread structural damage, underlying cognitive deterioration.^45^ The focus of the present study was mainly on cognitive status at follow‐up, as clinically relevant and practice measure of cognitive functioning. However, future research should additionally characterize the longitudinal changes in cognitive functioning.

Focusing on individual domains, volume loss of the DMN showed strongest associations with functioning of verbal and visuospatial memory and inhibition. The DMN itself appears to play a central role in memory retrieval and consolidation of extrinsic stimuli.^46^, ^47^ Previous functional MRI research on cognitive impairment found that regional atrophy of the DMN impact both memory and inhibitory control.^48^, ^49^ Considering cortical pathology in general, it has been suggested that more cortically oriented cognitive domains, that is, memory, might show higher vulnerability for decline when the disease progresses and cortical atrophy worsens.^50^ This might justify the indirect effects of CL volume via cortical volume loss predominantly in these domains. In contrast, executive functioning, including working memory and information processing speed, relies on efficient and precise transfer of information by short‐ and long‐range WM connections, and might therefore be more affected by WM abnormalities compared to other cognitive domains.^51^, ^52^

Along with the DMN, the found CI‐related volume losses of normal‐appearing cortex were evident in the VAN and SMN. Volume loss of the SMN additionally appeared the intermediate factor through which the effect of CL volume on cognition could be best explained. The SMN includes the bilateral sensorimotor cortices, which can predict physical performance.^18^ So, the CI‐related volume loss seen here might be reflective of overall disease burden and progression. However, recent insights have also indicated that the SMN might have specific and previously unknown cognitive connections, implicating a cognitive impact of the SMN in MS as well.^53^ The VAN includes the insular and anterior cingulate cortices, which are also known to be highly affected by MS‐related cortical focal demyelination and atrophy, that in turn can affect cognitive functioning.^54^ Parallel to these structural explanations of CI‐related network atrophy, previous functional MRI studies have shown a central role of neurodegeneration in the interplay between VAN and DMN in the development of cognitive impairment in people with MS.^19^, ^55^

Based on the mediation analysis, higher baseline CL volumes had a direct effect on worse cognitive functioning, with an indirect effect through cortical volume loss only for future cognitive worsening. This effect of CL volume on future functioning of specifically verbal and visuospatial memory and information processing speed could only be explained by an indirect effect through volume loss of normal‐appearing cortex, instead of by a direct effect of CL volume on cognitive functioning. The timing of CL formation relative to the cortical volume loss could not be explored given our study design, but remains an interesting avenue for future research. Still, these findings highlight the main driver of cognitive impairment is atrophy of nonlesional cortex, emphasizing the importance to inhibit and preferably prevent cortical atrophy to stop cognition from deteriorating. Unfortunately, neuroprotection as target of future therapeutic options remains crucially understudied. As of to date, current treatments do not successfully inhibit cognitive decline, possibly due to their focus on WM lesions only. Irreversible GM atrophy appears the strongest correlate of cognition,^1^ more so than WM lesion load,^21^ highlighted in our mediation analyses by the found independence of WM lesion volume. Of note, clinical and radiological evidence of active inflammation, such as clinical relapses and WM lesion formation during follow‐up, were not considered in our study. However, incorporating these measures could offer deeper insights into the role of active WM disease in the relationship between GM pathology and future cognitive outcomes. As CLs are known to differ in their underlying pathological profile and appear a potential substrate for irreversible atrophy, such treatments should also target these lesions, advocating clinical trials to study the efficacy of disease‐modifying therapies on the development and progression of CLs. For this, CLs need to be detectable in the clinical setting, however.

This study is not without limitations. While CL detection has been substantially improved by the use of DIR sequences compared to clinical FLAIR sequences,^56^ the majority of CLs still go undetected, in particular subpial lesions^41^. Therefore, the atrophied regions including normal‐appearing cortex might still have consisted of CLs, (partly) driving volume loss and thinning seen in normal‐appearing cortex and subsequent cognitive impairment. Also, in present study, we used regional *Z*‐scores in cortical volume and thickness measures to normalized the regional (physiological) variability in these MR measures. However, we acknowledge that this method may mask potentially interesting regional differences in the impact of lesional and normal‐appearing cortical volume or thickness on cognition. Future research should therefore explore the variations in the combined effects of CLs and regional cortical atrophy on cognition. Although we observed more pronounced cortical thinning in lesional cortex compared to normal‐appearing cortex, the lack of significant results for cognitive functioning related to the lesional cortex may have been influenced by the smaller number of regions affected by CLs, reducing statistical power. This challenge is intrinsic to CL analysis, as their distribution tends to be sparse and variable between patients. Future studies could address this by including participants based on their CL load, utilizing larger sample sizes and more targeted analyses to better capture the effects in lesional cortex. We also did not consider CL subtypes in our analyses. The impact of pure intracortical lesions versus lesions consisting of both WM and GM on cortical atrophy could differ and would be an interesting prospect for future studies. Finally, the results of our mediation analysis give rise to a novel perspective on the sequence of events occurring in the MS brain underlying cognitive decline. Unfortunately, we only had two time points at a 5‐year interval, which limited our ability to portray a definitive causal chain of events. Therefore, a longitudinal evaluation of the evolution of both WM and cortical demyelination and neurodegeneration would be worth exploring further.

In summary, this study showed that the relationship between MS‐related cortical volume loss at baseline and cognitive impairment at follow‐up was most likely driven by volume loss of normal‐appearing cortex, particularly in cortical networks related to cognition. This suggests that cortical atrophy can develop in the absence of focal cortical demyelination, with clear relevance for cognitive decline. Moreover, cortical volume loss in normal‐appearing cortex might potentially be a factor *in* the causal chain, standing between focal demyelination and the development of cognitive decline. These results warrant further studies explicitly looking into the interplay between focal and diffuse cortical damage for cognitive decline.

## Author Contributions

E.A.K. contributed to the conception and design of the study, acquisition and analysis of data, and drafting the manuscript/figures; M.v.D. contributed to the conception and design of the study, acquisition and analysis of data, and drafting a significant portion of the manuscript/figures; A.B., P.M.B., S.N., F.B., B.M.J.U., and M.D.S contributed to the acquisition and analysis of data; E.C.K. contributed to the conception and design of the study; I.K. contributed to the conception and design of the study, and the acquisition and analysis of data; M.M.S. contributed to the conception and design of the study, acquisition and analysis of data, and drafting a significant portion of the manuscript/figures.

## Conflicts of Interests

E.A.K. and A.B. report no conflicts of interest; M.v.D. has received a research grant from BMS; P.M.B. has received research support from the Dutch MS Research Foundation; S.N. has received research grants from Atara Biotherapeutics, Merck, and Biogen; F.B. is a steering committee or Data Safety Monitoring Board member for Biogen, Merck, ATRI/ACTC, and Prothena, serves as a consultant for Roche, Celltrion, Rewind Therapeutics, Merck, IXICO, Jansen, and Combinostics, has research agreements with Merck, Biogen, GE Healthcare, and Roche, and is co‐founder and shareholder of Queen Square Analytics LTD; B.M.J.U. has received consultancy fees from Immunic Therapeutics; M.D.S. has received research grants from Atara Biotherapeutics, Merck, and Biogen; E.C.K. has received consulting fees from EMD Serono, Genentech, INmune Bio, Myrobalan Therapeutics, OM1,and TG Therapeutics, and received research funds from Abbvie, Biogen, and Genentech; I.K has received research grants from LabEx TRAIL (Translational Research and Advanced Imaging Laboratory) and ARSEP (Fondation pour l'Aide à la Recherche sur la Sclérose En Plaques); M.M.S. serves on the editorial board of Neurology and Frontiers in Neurology and Multiple Sclerosis Journal, receives research support from the Dutch MS Research Foundation, Eurostars‐EUREKA, ARSEP, Amsterdam Neuroscience, MAGNIMS, and ZonMW and has served as a consultant for or received research support from EIP Pharma, Atara Biotherapeutics, Biogen, Celgene/Bristol Meyers Squibb, Genzyme, MedDay, and Merck.

## Supporting information


**Data S1.** Supporting Information.

## Data Availability

Tabular data supporting our findings are available from the corresponding author, upon reasonable request.
