# Supplementary material for: Cortical lesions impact cognitive decline in multiple sclerosis via volume loss of nonlesional cortex
Source: Ann Clin Transl Neurol. 2024 Dec 27;12(1):121–36. doi: 10.1002/acn3.52261 (PMC11752103; doi:10.1002/acn3.52261)
Supplement: Supplementary file 1 — Data S1. Supporting Information. [file ACN3-12-121-s001.docx]

**Methods Supplement**

**Clinical and cognitive assessments**

To evaluate physical disability at both time points in people with multiple sclerosis (MS), the Expanded Disability Status Scale (EDSS)^1^ was conducted by an experienced physician blinded to the imaging results. To evaluate cognitive functioning at baseline and 5-year follow-up, all participants underwent the Brief Repeatable Battery of Neuropsychological tests,^2^ as well as the Concept Shifting Test, the Stroop Color-Word Test, and the Memory Comparison Test. Tests were classified into seven predefined cognitive domains: attention [reflected by card 1 and 2 of the Stroop Color-Word Test^3^, intercept (time to complete 1-letter trial) of the Memory Comparison Test^4^ and Concept Shifting Test parts A and B^5^], information processing speed (Symbol Digit Modalities Test^6^), working memory (slope of the Memory Comparison Test^4^), visuospatial memory (the direct and delayed recall of the Spatial Recall Test^2^), verbal memory [the long-term storage (first trial and total), consistent long term retrieval, and delayed recall of the Selective Reminding Rest^7^], executive functioning (EF) – cognitive flexibility and verbal fluency (the shifting score of the Concept Shifting Test^5^ and Word List Generation Test^2^), and EF – inhibition (Stroop Color-Word Test interference^3^). Raw test scores of each cognitive domain were corrected for the effects of age, sex and education based on normative data for the Dutch general population. Corrected test scores were transformed into *Z*-scores based on the mean and standard deviation of the included healthy controls. At both time points, people with MS were considered cognitively-impaired if they performed ≤-2.0 SD below controls on two or more cognitive domains and mildly cognitively-impaired if they performed ≤-1.5 SD below controls on two of more cognitive domains. Remaining people with MS not fulfilling any of these criteria were classified as cognitively-preserved.^8,9^ Participants were excluded if *Z*-scores of cognitive domains were missing in 5 or more out of 7 cognitive domains at any of the two time points.

At baseline, 123 people with MS were considered CP, while 58 were categorized as MCI, and 49 as CI. At 5-year follow-up, 124 people with MS were classified as CP, 45 people as MCI, and 61 people as CI. Of the 123 CP-MS at baseline, 100 people with MS (81.3%) were still CP at 5-year follow-up, 14 people (11.4%) converted to MCI, and nine (7.3%) to CI. The 58 MCI-MS at baseline remained MCI at follow-up in 21 cases (36.2%), whereas 19 people (32.8%) converted to CI-MS; in 18 people (31.0%) cognitive functioning improved to the level of CP-MS. As such, 33 of 49 CI-MS at baseline (67.3%) remained CI at follow-up, whereas in 16 people (32.6%) their cognitive performance improved to the level of CP (*N*=6; 12.2%) or MCI (*N*=10; 20.4%).

**References**

1. Kurtzke JF. Rating neurologic impairment in multiple sclerosis: an expanded disability status scale (EDSS). *Neurology*. Nov 1983;33(11):1444-52. doi:10.1212/wnl.33.11.1444

2. Rao S. A manual for brief repeatable battery of the neuropsychological tests in multiple sclerosis. Milwaukee:  Medical College of Wisconsin; 1990.

3. Van der Elst W, Van Boxtel MP, Van Breukelen GJ, Jolles J. Detecting the significance of changes in performance on the Stroop Color-Word Test, Rey's Verbal Learning Test, and the Letter Digit Substitution Test: the regression-based change approach. *J Int Neuropsychol Soc*. Jan 2008;14(1):71-80. doi:10.1017/S1355617708080028

4. Van Der Elst W, Van Boxtel MP, Van Breukelen GJ, Jolles J. Assessment of information processing in working memory in applied settings: the paper and pencil memory scanning test. *Psychol Med*. Sep 2007;37(9):1335-44. doi:10.1017/S0033291707000360

5. Van der Elst W, Van Boxtel MP, Van Breukelen GJ, Jolles J. The Concept Shifting Test: adult normative data. *Psychol Assess*. Dec 2006;18(4):424-32. doi:10.1037/1040-3590.18.4.424

6. Smith A. Symbol Digits Modalities Test. Western Psychological Services,  Los Angeles; 1982.

7. Buschke H, Fuld PA. Evaluating storage, retention, and retrieval in disordered memory and learning. *Neurology*. Nov 1974;24(11):1019-25. doi:10.1212/wnl.24.11.1019

8. Eijlers AJC, Wink AM, Meijer KA, Douw L, Geurts JJG, Schoonheim MM. Reduced Network Dynamics on Functional MRI Signals Cognitive Impairment in Multiple Sclerosis. *Radiology*. 08 2019;292(2):449-457. doi:10.1148/radiol.2019182623

9. Schoonheim MM, Hulst HE, Brandt RB, et al. Thalamus structure and function determine severity of cognitive impairment in multiple sclerosis. *Neurology*. Feb 24 2015;84(8):776-83. doi:10.1212/WNL.0000000000001285

**Supplementary Table 1. Allocation of cortical atlas regions into functional networks**

| Functional network | Brainnetome atlas regions |
| --- | --- |
| Default mode | Left and right dorsolateral area 8; Left and right lateral area 9; Left and right medial area 9; Left and right medial area 10; Left ventrolateral area 8; Left lateral area10; Left caudal area 45; Left rostral area 45; Left ventral area 44; Left and right medial area 14; Left and right orbital area 12/47; Left and right lateral area 12/47; Left rostral area 22; Left and right caudal area 21; Left and right rostral area 21; Left and right anterior superior temporal sulcus; Left intermediate lateral area 20; Left area TL (lateral PPHC, posterior parahippocampal gyrus); Left and right rostroposterior superior temporal sulcus; Left caudoposterior superior temporal sulcus; Left caudal area 40(PFm); Left and right rostroventral area 39(PGa); Left dorsomedial parietooccipital sulcus (PEr); Left and right area 31 (Lc1); Left ventral agranular insula; Left and right dorsal area 23; Right rostroventral area 24; Left pregenual area 32; Left and right ventral area 23; Left and right subgenual area 32; left and right hippocampus |
| Dorsal attention | Left and right dorsolateral area 6; Left ventrolateral area 6; Left and right caudal dorsolateral area 6; Left and right caudal ventrolateral area 6; Left and right dorsolateral area37; Left and right extreme lateroventral area37; Left and right ventrolateral area 37; Left lateroventral area37; Left and right rostral area 7; Left and right caudal area 7; Left and right lateral area 5; Left and right intraparietal area 7 (hIP3); Right caudal area 39 (PGp); Left and right rostrodorsal area 40 (PFt); Right medial area 7 (PEp); Right medial area 5 (PEm) |
| Frontoparietal | Right dorsal area 9/46; Left and right inferior frontal junction; Left and right area 46; Left and right ventral area 9/46; Right ventrolateral area 8; Right ventrolateral area 6; Right lateral area10; Left and right dorsal area 44; Left and right inferior frontal sulcus; Right caudal area 45; Right rostral area 45; Right ventral area 44; Left and right caudolateral of area 20; Left and right rostrodorsal area 39 (Hip3); Right caudal area 40 (PFm); Left medial area 7 (PEp); Left rostroventral area 24 |
| Limbic | Left and right lateral area 11; Left and right medial area 11; Left and right area 13; Left and right medial area 38; Left and right lateral area 38; Left and right intermediate ventral area 20; Left and right rostral area 20; Right intermediate lateral area 20; Left and right caudoventral of area 20; Left and right rostroventral area 20; Left and right rostral area 35/36; Left and right caudal area 35/36; Left and right area 28/34 (EC, entorhinal cortex); Left and right area TI (temporal agranular insular cortex) |
| Sensorimotor | Left and right medial area 6; Left and right area 4 (head and face region); Left and right area 4 (upper limb region); Left and right area 4 (trunk region); Left and right area1/2/3 (lower limb region); Left and right area 4 (lower limb region); Left and right area 41/42; Left and right TE1.0 and TE1.2; Left and right caudal area 22; Right rostral area 22; Left and right postcentral area 7; Left and right area 1/2/3 (upper limb, head and face region); Left and right area 1/2/3; Left and right area 2; Left and right area1/2/3 (trunk region); Left and right hypergranular insula; Left and right dorsal granular insula |
| Ventral attention | Left and right medial area 8; Left dorsal area 9/46; Left and right opercular area 44; Left and right area 4 (tongue and larynx region); Right caudoposterior superior temporal sulcus; Left and right rostroventral area 40 (PFop); Left medial area 5 (PEm); Right ventral agranular insula; Left and right dorsal agranular insula; Left and right ventral dysgranular and granular insula; Left and right dorsal dysgranular insula; Right pregenual area 32; Left and right caudodorsal area 24; Left and right caudal area 23 |
| Visual | Left and right medioventral area37; Right lateroventral area37; Right area TL (lateral PPHC, posterior parahippocampal gyrus); Left and right area TH (medial PPHC); Left caudal area 39 (PGp); Right dorsomedial parietooccipital sulcus (PEr); Left and right caudal lingual gyrus; Left and right rostral cuneus gyrus; Left and right caudal cuneus gyrus; Left and right rostral lingual gyrus; Left and right ventromedial parietooccipital sulcus; Left and right middle occipital gyrus; Left and right area V5/MT+; Left and right occipital polar cortex; Left and right inferior occipital gyrus; Left and right medial superior occipital gyrus; Left and right lateral superior occipital gyrus |

Allocation of 210 Brainnetome atlas^1^ regions, including the hippocampi of FSL FIRST segmentation, into seven functional networks based on the Yeo atlas^2^.

^1^Fan L, Li H, Zhuo J, et al. The Human Brainnetome Atlas: A New Brain Atlas Based on Connectional Architecture. *Cereb Cortex*. 08 2016;26(8):3508-26. doi:10.1093/cercor/bhw157

^2^Yeo BT, Krienen FM, Sepulcre J, et al. The organization of the human cerebral cortex estimated by intrinsic functional connectivity. *J Neurophysiol*. Sep 2011;106(3):1125-65. doi:10.1152/jn.00338.2011

**Supplementary Table 2. Neuropsychological functioning in MS participants**

|  | MS  N=230 | CP-MS  N=123 | MCI-MS  N=58 | CI-MS  N=49 | | *Group differences* | |
| --- | --- | --- | --- | --- | --- | --- | --- |
|  |  |  |  |  |  | *F-test, P-value* | |
| Neuropsychological functioning at baseline, as Z-score, *Mean ± SD* | | | | | | | |
| Average cognition  Attention  EF – inhibition  EF – CF and verbal fluency  IPS  Verbal memory  Visuospatial memory  Working memory | -0.64 (0.72)  -0.87 (1.03)  -0.32 (1.14)  -0.44 (1.12)  -1.06 (1.21)  -0.64 (1.10)  -0.68 (1.20)  -0.44 (1.39) | -0.18 (0.45)  -0.37 (0.62)  0.09 (0.95)  -0.03 (0.76)  -0.47 (0.98)  -0.15 (0.91)  -0.23 (1.04)  -0.08 (1.10) | -0.88 (0.31)  -1.15 (0.81)  -0.69 (1.17)  -0.31 (0.73)  -1.52 (0.85)  -0.97 (0.99)  -1.08 (1.10)  -0.42 (1.33) | -1.53 (0.65)  -1.82 (1.28)  -0.91 (1.18)  -1.63 (1.40)  -2.00 (1.29)  -1.53 (0.96)  -1.35 (1.25)  -1.35 (1.71) | | *F(2,227)=154.10, P=****5.27⋅10^-43^****^a,b,c^*  *F(2,225)=54.86, P=****3.92⋅10^-20^****^a,b,c^*  *F(2,218)=19.37, P=****1.81⋅10^-8^****^a,c^*  *F(2,227)=53.27, P=****1.08⋅10^-19^****^b,c^*  *F(2,226)=46.80, P=****9.84⋅10^-18^****^a,b,c^*  *F(2,225)=41.18, P=****5.75⋅10^-16^****^a,b,c^*  *F(2,227)=23.14, P=****7.18⋅10^-10^****^a,c^*  *F(2,220)=15.95, P=****3.39⋅10^-7^****^b,c^* | |
|  | **MS**  **N=230** | **CP-MS**  **N=124** | **MCI-MS**  **N=45** | | **CI-MS**  **N=61** | | ***Group differences*** |
|  |  |  |  |  |  |  | *F-test, P-value* |
| Neuropsychological functioning at 5-year follow-up, as *Z*-score, *Mean ± SD* | | | | | | | |
| Average cognition  Attention  EF – inhibition  EF – CF and verbal fluency  IPS  Verbal memory  Visuospatial memory  Working memory | -0.71 (0.74)  -0.52 (1.00)  -0.27 (1.48)  -0.56 (0.98)  -1.20 (1.13)  -0.96 (1.10)  -1.01 (1.30)  -0.42 (1.32) | -0.29 (0.54)  -0.08 (0.75)  0.26 (1.39)  -0.27 (0.78)  -0.69 (1.03)  -0.56 (0.91)  -0.62 (1.24)  -0.04 (1.05) | -0.89 (0.43)  -0.72 (0.84)  -0.50 (1.12)  -0.53 (0.85)  -1.59 (0.81)  -1.05 (1.07)  -1.31 (1.16)  -0.53 (1.33) | -1.55 (0.67)  -1.39 (1.09)  -1.36 (1.48)  -1.31 (1.17)  -2.03 (1.03)  -1.85 (1.03)  -1.62 (1.32)  -1.23 (1.52) | | *F(2,227)=97.03, P=****3.52⋅10^-31^****^a,b,c^*  *F(2,225)=43.20, P=****1.33⋅10^-16^****^a,b,c^*  *F(2,221)=25.06, P=****1.56⋅10^-10^****^a,b,c^*  *F(2,227)=23.79, P=****4.17⋅10^-10^****^b,c^*  *F(2,226)=39.16, P=****2.49⋅10^-15^****^a,c^*  *F(2,226)=30.68, P=****1.63⋅10^-12^****^a,b,c^*  *F(2,227)=13.77, P=****2.00⋅10^-16^****^a,c^*  *F(2,220)=16.12, P=****2.92⋅10^-7^****^a,b,c^* | |

Baseline and 5-year cognitive performance of cognitive groups at baseline. After correction for age, sex and education, cognitive domain scores were transformed into *Z*-scores based on neuropsychological assessment data of healthy controls at the respective time point. Mean *Z*-scores with standard deviation (SD) are reported in the table. Significant *P*-values are marked in bold.

MS = multiple sclerosis; CP = cognitively-preserved; MCI = mildly cognitively-impaired; CI = cognitively-impaired; IPS = information processing speed; EF = executive functioning; CF = cognitive flexibility.
^a^Significant Bonferroni-corrected post-hoc difference (*P*<0.05) between CP-MS and MCI-MS

^b^Significant Bonferroni-corrected post-hoc difference (*P*<0.05) between MCI-MS and CI-MS

^c^Significant Bonferroni-corrected post-hoc difference (*P*<0.05) between CP-MS and CI-MS

**Supplementary Table 3. Demographics of people with multiple sclerosis with and without cortical lesions**

|  | MS with CLs  N=199 | MS without CLs  N=31 | *MS with vs without CLs* |
| --- | --- | --- | --- |
|  |  |  | *Test statistic, P-value* |
| Demographics |  |  |  |
| Age at baseline (years) | 48.1 (10.9) | 44.2 (10.7) | *t(228)=-1.89, P=0.059* |
| Sex (female), *N* (%) | 134 (67.3) | 21 (67.7) | *X^2^(1)=0.002, P=0.964* |
| Level of education (high^a^), *N (%)* | 91 (45.7) | 10 (32.3) | *X^2^(1)=1.98, P=0.160* |
| MS characteristics |  |  |  |
| MS subtype (RR/SP/PP), *N* (%) | 151 (75.9)/33 (16.6)/15 (7.5) | 28 (90.3)/0 (0)/3 (9.7) | *X^2^(2)=6.02, P=****0.049*** |
| EDSS^b^ | 3.0 [2.0] | 3.0 [1.5] | *U=2674.5, P=0.231* |
| Disease duration (years) | 12.2 (6.7) | 8.7 (5.6) | *t(44.9)=-3.05, P=****0.004*** |
| Medication (yes), *N (%)*  *First-line treatment*  *Second-line treatment* | 73 (36.7)  59 (29.7)  14 (7.0) | 11 (35.5)  9 (29.0)  2 (6.5) | *X^2^(1)=0.02, P=0.897* |
| MRI characteristics | |  |  |
| NGM volume | 0.42 (0.03) | 0.43 (0.02) | *t(228)=1.51, P=0.132* |
| NDGM volume | 0.034 (0.003) | 0.036 (0.002) | *t(228)=2.88, P=****0.004*** |
| NWM volume | 0.28 (0.02) | 0.29 (0.02) | *t(228)=1.40, P=0.162* |
| WM lesion volume (mL)^b,c^ | 10.32 [13.50] | 3.77 [4.29] | *t(228)=6.05, P=****5.96⋅10^-9^*** |

Demographics of people with multiple sclerosis (MS) with and without cortical lesions (CLs) are reported as Mean (SD) unless specific otherwise. Significant *P*-values are marked in bold.

^a^Level of education was dichotomized and defined as high educational level corresponding to ≥6 on the Dutch Verhage scale [Verhage F. Intelligence and Age in a Dutch Sample. 8(4):238-245].

^b^Median [interquartile range]

^c^Comparison analyses performed on log-scale.

FU = follow-up; RR = relapsing remitting; SP = secondary progressive; PP = primary progressive; EDSS = expanded disability status scale; NGM = normalized total gray matter volume; NDGM = normalized deep gray matter volume; NWM = normalized white matter.

**Supplementary Table 4. Distribution of cortical lesions**

|  | MS  N=230 | CP-MS  N=123 | MCI-MS  N=58 | CI-MS  N=49 | *CP-MS versus MCI-MS^a^* | *CP-MS versus CI-MS^a^* | *MCI-MS versus CI-MS^a^* |
| --- | --- | --- | --- | --- | --- | --- | --- |
|  |  |  |  |  | *OR (95% confidence interval), P-value* | *OR (95% confidence interval), P-value* | *OR (95% confidence interval), P-value* |
| Global CL characteristics | | |  |  |  |  |  |
| *Presence of ≥ 1 CL* | 199 (86.5) | 107 (87.0) | 47 (81.0) | 45 (91.8) | *0.67 (0.29;1.58), P=0.361* | *1.57 (0.49;5.09), P=0.452* | *2.34 (0.68;8.08), P=0.179* |
| *CL volume, mL in x10^-3^* | 124.5 [262.3] | 85.0 [202.0] | 148.0 [319.5] | 267.0 [533.5] | *1.06 (0.91;1.23), P=0.458* | *1.19 (0.99;1.43), P=0.065* | *1.12 (0.91;1.38), P=0.273* |
| Functionally related areas consisting of regions with CL | | | | | |  |  |
| DMN  *Presence of ≥ 1 CL*  *CL volume, mL in x10^-3^* | 163 (70.9)  21.0 [72.0] | 84 (68.3)  15.0 [56.0] | 41 (70.7)  29.0 [73.5] | 38 (77.6)  49.0 [128.5] | *1.15 (0.58;2.29), P=0.695*  *1.10 (0.94;1.29), P=0.243* | *1.46 (0.66;3.20), P=0.349*  *1.22 (1.02;1.47), P=0.027* | *1.27 (0.52;3.10), P=0.601*  *1.11 (0.91;1.36), P=0.300* |
| DAN  *Presence of ≥ 1 CL*  *CL volume, mL in x10^-3^* | 129 (56.1)  7.0 [37.3] | 62 (50.4)  2.0 [25.0] | 35 (60.3)  15.0 [51.8] | 32 (65.3)  17.0 [56.0] | *1.59 (0.83;3.04), P=0.159*  *1.15 (0.97;1.35), P=0.110* | *1.79 (0.88;3.62), P=0.108*  *1.20 (1.00;1.43), P=0.048* | *1.12 (0.50;2.52), P=0.780*  *1.05 (0.86;1.28), P=0.458* |
| FPN  *Presence of ≥ 1 CL*  *CL volume, mL in x10^-3^* | 111 (48.3)  0 [24.0] | 53 (43.1)  0.0 [19.0] | 28 (48.3)  0.0 [24.0] | 30 (61.2)  7.0 [66.0] | *1.28 (0.68;2.43), P=0.442*  *1.05 (0.88;1.25), P=0.611* | *1.96 (0.98;3.92), P=0.058*  *1.21 (1.01;1.45), P=0.043* | *1.53 (0.69;3.36), P=0.293*  *1.15 (0.94;1.42), P=0.177* |
| Limbic network  *Presence of ≥ 1 CL*  *CL volume, mL in x10^-3^* | 168 (73.0)  26.5 [84.5] | 91  (74.0)  25.0 [62.0] | 41 (70.7)  26.5 [83.3] | 36 (73.5)  61.0 [146.0] | *0.90 (0.44;1.84), P=0.777*  *1.02 (0.87;1.19), P=0.838* | *0.88 (0.41;1.93), P=0.756*  *1.10 (0.93;1.32), P=0.273* | *0.98 (0.41;2.36), P=0.963*  *1.09 (0.89;1.33), P=0.421* |
| SMN  *Presence of ≥ 1 CL*  *CL volume, mL in x10^-3^* | 142 (61.7)  11.0 [42.3] | 65 (52.8)  3.0 [23.0] | 41 (70.7)  14.0 [54.3] | 36 (73.5)  27.0 [61.0] | *2.19 (1.12;4.28), P=0.022*  *1.24 (1.05;1.47), P=0.011* | *2.55 (1.22;5.37), P=0.013*  *1.30 (1.08;1.55), P=****0.005*** | *1.17 (0.49;2.77), P=0.725*  *1.04 (0.85;1.28), P=0.691* |
| VAN  *Presence of ≥ 1 CL*  *CL volume, mL in x10^-3^* | 139 (60.4)  9.0 [49.5] | 67 (54.5)  3.0 [39.0] | 39 (67.2)  21.0 [59.0] | 33 (67.3)  28.0 [106.5] | *1.83 (0.94;3.56), P=0.076*  *1.20 (1.02;1.41), P=0.029* | *1.60 (0.78;3.27), P=0.198*  *1.21 (1.01;1.43), P=0.034* | *0.88 (0.38;2.01), P=0.754*  *1.01 (0.83;1.23), P=0.458* |
| Visual network  *Presence of ≥ 1 CL*  *CL volume, mL in x10^-3^* | 60 (26.1)  0.0 [1.0] | 22 (17.9)  0.0 [0.0] | 20 (34.5)  0.0 [12.0] | 18 (36.7)  0.0 [14.0] | *2.49 (1.21;5.10), P=0.013*  *1.48 (1.17;1.86), P=****9.55⋅10^-4^*** | *2.66 (1.25;5.67), P=0.011*  *1.41 (1.11;1.80), P=****0.005*** | *1.07 (0.48;2.40), P=0.871*  *0.96 (0.76;1.20), P=0.701* |

Distribution of cortical lesions (CL) across cortical networks in people with multiple sclerosis (MS), divided in three cognitive subgroups: cognitively-preserved (CP), mildly cognitively-impaired (MCI) and cognitively-impaired (CI) patients. Volumes of CL are reported as *Median [interquartile range]*, and were log(x+1)-transformed before group comparisons. Presence of at least one CL is shown as *N (%)*. Raw unadjusted *P*-values are shown. Significant *P*-values surviving Bonferroni correction (*P*<0.007) are marked in bold.

^a^Volumes of CLs and presence of at least one CL were analyzed with the use of logistic regression for cognitive subgroups, adjusting for age, sex, and high level of education. Odds ratios (OR) with 95% confidence intervals and corresponding *P*-values are reported.

DMN = default mode network; DAN = dorsal attention network; FPN = frontoparietal network; SMN = sensorimotor network; VAN = ventral attention network.

**Supplementary Table 5. Baseline cortical gray matter volumetrics in cognitive subgroups at baseline**

|  | CP-MS  N=123 | MCI-MS  N=58 | CI-MS  N=49 | *CP-MS versus MCI-MS^a^* | *CP-MS versus CI-MS^a^* | *MCI-MS versus CI-MS^a^* |
| --- | --- | --- | --- | --- | --- | --- |
|  |  |  |  | *OR (95% confidence interval), P-value* | *OR (95% confidence interval), P-value* | *OR (95% confidence interval), P-value* |
| Global cortical volume measures, as Z-score, *Mean (SD)* | | | | |  |  |
| Thickness  NA cortex  Lesional cortex | -0.31 (0.55)  -0.30 (0.53)  -0.58 (0.78) | -0.44 (0.56)  -0.41 (0.53)  -0.77 (0.71) | -0.72 (0.76)  -0.68 (0.74)  -0.92 (1.04) | *0.58 (0.31;1.07), P=0.079*  *0.86 (0.31;2.44), P=0.782*  *0.76 (0.38;1.52), P=0.440* | *0.35 (0.18;0.67), P=****0.001***  *0.31 (0.10;0.93), P=0.036*  *1.22 (0.58;2.59), P=0.598* | *0.61 (0.30;1.21), P=0.156*  *0.36 (0.10;1.24), P=0.104*  *1.61 (0.68;3.80), P=0.280* |
| Norm. Volume  NA cortex  Lesional cortex | -0.07 (0.39)  -0.07 (0.38)  -0.04 (0.72) | -0.17 (0.36)  -0.16 (0.36)  -0.12 (0.55) | -0.26 (0.46)  -0.25 (0.45)  -0.22 (0.84) | *0.40 (0.16;1.00), P=0.049*  *0.28 (0.08;1.01), P=0.051*  *1.32 (0.65;2.67), P=0.449* | *0.34 (0.13;0.90), P=0.029*  *0.31 (0.08;1.16), P=0.082*  *1.27 (0.60;2.70), P=0.075* | *0.85 (0.30;2.41), P=0.762*  *1.10 (0.25;4.80), P=0.896*  *0.97 (0.41;2.31), P=0.941* |

Cortical thickness and normalized cortical volume of cognitively-preserved (CP), mildly cognitively-impaired (MCI) and cognitively-impaired (CI) people with multiple sclerosis (MS) at baseline are shown for the entire cortex, as well as for normal-appearing (NA) and lesional cortex separately. Significant *P*-values surviving Bonferroni correction for multiple testing (*P*<0.025) are marked in bold.

^a^Logistic regression model of global cortical volumetrics for cognitive classification at baseline, adjusted for age, sex and high level of education. Subanalyses of NA and lesional cortex were performed by including both variables as independent variables in the logistic regression model, in which only people with MS with cortical lesions were included, eliminating potential bias from people with MS without cortical lesions. Odds ratios (OR) with 95% confidence intervals and corresponding *P*-values are reported.

**Supplementary Table 6. Baseline cortical thickness and volume among cognitive subgroups at 5-year follow-up**

|  | CP-MS  N=124 | MCI-MS  N=45 | CI-MS  N=61 | *CP-MS versus MCI-MS^a^* | *CP-MS versus CI-MS^a^* | | *MCI-MS versus CI-MS^a^* |
| --- | --- | --- | --- | --- | --- | --- | --- |
|  |  |  |  | *OR (95% confidence interval), P-value* | *OR (95% confidence interval), P-value* | | *OR (95% confidence interval), P-value* |
| Global cortical volume measures, as *Z*-scores, *Mean (SD)* | | | | |  | |  |
| *Thickness*  *NA cortex*  *Lesional cortex* | -0.26 (0.49)  -0.25 (0.49)  -0.53 (0.72) | -0.51 (0.68)  -0.47 (0.64)  -0.86 (0.91) | -0.71 (0.69)  -0.68 (0.67)  -0.94 (0.94) | *0.50 (0.23;1.07), P=0.072*  *0.56 (0.15;2.06), P=0.383*  *0.85 (0.36;2.03), P=0.717* | *0.35 (0.16;0.76), P=****0.008***  *0.21 (0.06;0.82), P=****0.025***  *1.35 (0.56;3.26), P=0.509* | | *0.71 (0.34;1.47), P=0.357*  *0.38 (0.10;1.46), P=0.157*  *1.58 (0.63;3.95), P=0.327* |
| *Norm. volume*  *NA cortex*  *Lesional cortex* | -0.01 (0.33)  -0.01 (0.33)  0.09 (0.75) | -0.23 (0.39)  -0.22 (0.38)  -0.30 (0.57) | -0.32 (0.45)  -0.32 (0.46)  -0.34 (0.61) | *0.16 (0.05;0.56), P=****0.004***  *0.21 (0.04;1.06), P=0.059*  *0.59 (0.25;1.44), P=0.250* | *0.08 (0.02;0.31), P=****1.81⋅10^-4^***  *0.10 (0.02;0.57), P=****0.009***  *0.63 (0.26;1.53), P=0.306* | | *0.53 (0.17;1.63), P=0.265*  *0.51 (0.11;2.44), P=0.396*  *1.06 (0.40;2.81), P=0.910* |
| Network-level cortical volume measures, as *Z*-scores, *Mean (SD)* | | | | | | |  |
| DMN | |  |  |  |  | |  |
| *Norm. volume*  *NA cortex*  *Lesional cortex* | -0.07 (0.40)  -0.08 (0.40)  0.02 (0.78) | -0.32 (0.45)  -0.31 (0.44)  -0.35 (0.74) | -0.45 (0.50)  -0.44 (0.50)  -0.46 (0.92) | *0.23 (0.08;0.68), P=0.008*  *0.23 (0.06;0.79), P=0.020*  *0.83 (0.45;1.54), P=0.555* | *0.12 (0.04;0.36), P=****1.65⋅10^-4^***  *0.13 (0.03;0.46), P=****0.002***  *0.87 (0.45;1.67), P=0.671* | | *0.51 (0.18;1.42), P=0.197*  *^b^*  *^b^* |
| DAN | |  |  |  |  | |  |
| *Norm. volume*  *NA cortex*  *Lesional cortex* | -0.01 (0.40)  -0.02 (0.39)  0.11 (1.03) | -0.15 (0.44)  -0.15 (0.42)  -0.12 (1.06) | -0.27 (0.51)  -0.26 (0.50)  -0.42 (0.84) | *0.48 (0.18;1.29), P=0.143*  *^b^*  *^b^* | *0.23 (0.08;0.63), P=****0.004***  *0.21 (0.05;0.89), P=0.035*  *0.68 (0.36;1.28), P=0.232* | | *0.48 (0.17;1.32), P=0.155*  *^b^*  *^b^* |
| FPN | |  |  |  |  | |  |
| *Norm. volume*  *NA cortex*  *Lesional cortex* | -0.01 (0.40)  -0.01 (0.40)  -0.03 (0.89) | -0.10 (0.51)  -0.10 (0.51)  -0.05 (0.80) | -0.27 (0.49)  -0.29 (0.49)  -0.32 (0.80) | *0.83 (0.30;2.25), P=0.711*  *^b^*  *^b^* | *0.27 (0.10;0.74), P=0.011*  *0.34 (0.07;1.69), P=0.188*  *0.41 (0.15;1.10), P=0.076* | | *0.32 (0.11;0.95), P=0.041*  *0.40 (0.08;2.11), P=0.281*  *0.47 (0.17;1.34), P=0.160* |
| Limbic network | |  |  |  |  | |  |
| *Norm. volume*  *NA cortex*  *Lesional cortex* | -0.08 (0.47)  -0.08 (0.45)  -0.03 (0.97) | -0.36 (0.46)  -0.35 (0.47)  -0.60 (0.89) | -0.37 (0.57)  -0.34 (0.58)  -0.50 (0.70) | *0.29 (0.13;0.68), P=****0.004***  *0.29 (0.09;0.90), P=0.032*  *0.71 (0.35;1.41), P=0.327* | *0.29 (0.12;0.69), P=****0.005***  *0.34 (0.11;1.04), P=0.059*  *0.84 (0.43;1.62), P=0.600* | | *1.00 (0.43;2.29), P=0.990*  *^b^*  *^b^* |
| SMN | |  |  |  |  | |  |
| *Norm. volume*  *NA cortex*  *Lesional cortex* | 0.07 (0.49)  0.06 (0.48)  0.23 (0.91) | -0.23 (0.55)  -0.23 (0.54)  -0.22 (0.84) | -0.31 (0.62)  -0.31 (0.63)  -0.41 (1.16) | *0.23 (0.10;0.56), P=****0.001***  *0.16 (0.05;0.56), P=****0.004***  *0.92 (0.46;1.84), P=0.805* | *0.19 (0.08;0.47), P=****3.25⋅10^-4^***  *0.14 (0.04;0.51), P=****0.003***  *0.75 (0.37;1.54), P=0.439* | | *0.82 (0.38;1.77), P=0.606*  *^b^*  *^b^* |
| VAN | |  |  |  |  | |  |
| *Norm. volume*  *NA cortex*  *Lesional cortex* | -0.01 (0.43)  -0.02 (0.43)  0.17 (0.85) | -0.26 (0.55)  -0.25 (0.56)  -0.52 (0.85) | -0.30 (0.50)  -0.32 (0.54)  -0.31 (0.64) | *0.28 (0.11;0.69), P=****0.006***  *0.29 (0.09;0.96), P=0.042*  *0.50 (0.24;1.02), P=0.056* | *0.24 (0.09;0.62), P=****0.003***  *0.16 (0.04;0.61), P=****0.007***  *0.84 (0.39;1.84), P=0.670* | | *0.88 (0.35;2.17), P=0.774*  *^b^*  *^b^* |
| Visual network | |  |  |  |  |  |  |
| *Norm. volume*  *NA cortex*  *Lesional cortex* | 0.05 (0.46)  0.05 (0.47)  0.10 (0.79) | -0.15 (0.44)  -0.15 (0.43)  -0.08 (0.89) | -0.26 (0.52)  -0.26 (0.53)  -0.27 (0.67) | *0.46 (0.20;1.07), P=0.072*  *^b^*  *^b^* | *0.28 (0.12;0.68), P=****0.005***  *0.33 (0.05;2.37), P=0.272*  *0.55 (0.17;1.82), P=0.328* | | *0.61 (0.24;1.51), P=0.280*  *^b^*  *^b^* |

Normalized cortical volume and cortical thickness of cognitively-preserved (CP), mildly cognitively-impaired (MCI) and cognitively-impaired (CI) people with multiple sclerosis (MS) at baseline are shown for the entire cortex, as well as for normal-appearing (NA) and lesional cortex separately. Significant *P*-values surviving Bonferroni correction for multiple testing (*P*<0.025 for global measures, *P*<0.007 for network measures) are marked in bold.

^a^Logistic regression model of cortical volumetrics per network for cognitive classification at 5-year follow-up, adjusted for age, sex, high level of education and cognitive classification as baseline. Subanalyses of NA and lesional cortex were performed by including both variables as independent variables in the logistic regression model, in which only people with MS with cortical lesions were included, eliminating potential bias from people with MS without cortical lesions. Odds ratios (OR) with 95% confidence intervals and corresponding *P*-values are reported.

^b^Regression not performed due to non-significant main result.

DMN = default mode network; DAN = dorsal attention network; FPN = frontoparietal network; SMN = sensorimotor network; VAN = ventral attention network.

**Supplementary Table 7. Results of mediation analyses for cortical volume**

|  | | Normalized volume |
| --- | --- | --- |
|  | | *est ML (95% confidence interval), P-value* |
| Average cognition | Direct (c)  Indirect (a*b)  Total (c+(a*b)) | *-0.02 (-0.07;0.02), P=0.293*  *-0.05 (-0.07;-0.02), P=****9.98⋅10^-5^***  *-0.07 (-0.12;-0.02), P=****0.003*** |
| EF – Inhibition | Direct (c)  Indirect (a*b)  Total (c+(a*b)) | *-0.05 (-0.20;0.10), P=0.549*  *-0.07 (-0.13;-0.02), P=0.011*  *-0.12 (-0.27;0.03), P=0.106* |
| Information processing speed | Direct (c)  Indirect (a*b)  Total (c+(a*b)) | *-0.08 (-0.17;0.01), P=0.089*  *-0.03 (-0.07;-0.00), P=0.035*  *-0.11 (-0.20;-0.03), P=****0.010*** |
| Verbal memory | Direct (c)  Indirect (a*b)  Total (c+(a*b)) | *-0.05 (-0.14;0.03), P=0.241*  *-0.07 (-0.10;-0.03), P=****0.001***  *-0.12 (-0.20;-0.03), P=****0.007*** |
| Visuospatial memory | Direct (c)  Indirect (a*b)  Total (c+(a*b)) | *-0.06 (-0.19;0.07), P=0.364*  *-0.10 (-0.16;-0.04), P=****0.001***  *-0.16 (-0.29;-0.03), P=0.016* |

Outcome variables (cognitive functioning at 5-year follow-up) are shown on the left, divided into three effect estimates: direct (c), indirect (a*b) and total effect (c+(a*b)). The predictor variable is the log(x+1)-transformed cortical lesion volume, and mediator variable is the mean *Z*-score of normalized cortical volume relative to the included healthy volunteers. Included confounders are age, sex, high level of education, and baseline cognitive functioning. Estimates are reported as maximum likelihood (*est ML*) with 95% confidence interval and *P*-value. Significant *P*-values surviving Bonferroni correction for multiple testing (*P*<0.010) are marked in bold. EF = executive functioning

**Supplementary Table 8. Results of mediation analyses for cortical volume in lesional and normal-appearing cortex**

|  | | Normalized volume |
| --- | --- | --- |
|  | | *est ML (95% confidence interval), P-value* |
| Average cognition | Direct (c)  Indirect 1 (a*b)  Indirect 2 (d*e)  Total (c+(a*b)+(d*e)) | *-0.02 (-0.07;0.02), P=0.315*  *-0.01 (-0.02;0.01), P=0.416*  *-0.04 (-0.06;-0.02), P=****2.70⋅10^-4^***  *-0.07 (-0.12;-0.02), P=****0.003*** |
| Information processing speed | Direct (c)  Indirect 1 (a*b)  Indirect 2 (d*e)  Total (c+(a*b)+(d*e)) | *-0.09 (-0.18;0.00), P=0.062*  *0.03 (-0.00;0.06), P=0.060*  *-0.05 (-0.09;-0.02), P=****0.004***  *-0.11 (-0.20;-0.03), P=****0.010*** |
| Verbal memory | Direct (c)  Indirect 1 (a*b)  Indirect 2 (d*e)  Total (c+(a*b)+(d*e)) | *-0.05 (-0.14;0.04), P=0.268*  *-0.01 (-0.03;0.02), P=0.612*  *-0.06 (-0.10;-0.02), P=****0.001***  *-0.12 (-0.20;-0.03), P=****0.007*** |
| Visuospatial memory | Direct (c)  Indirect 1 (a*b)  Indirect 2 (d*e)  Total (c+(a*b)+(d*e)) | *-0.06 (-0.20;0.07), P=0.365*  *-0.00 (-0.04;0.04), P=0.979*  *-0.10 (-0.15;-0.04), P=****0.001***  *-0.16 (-0.29;-0.03), P=0.016* |

Outcome variables (cognitive functioning at 5-year follow-up) are shown on the left, divided into four effect estimates: direct (c), indirect 1 (a*b), indirect 2 (d*e) and total effect (c+(a*b)+(d*e)). Predictor variable is log(x+1)-transformed cortical lesion volume, and mediator variables are mean *Z*-scores of the normalized cortical volume in lesional cortex (indirect 1) and normal-appearing cortex (indirect 2). Included confounders are age, sex, high level of education, and baseline cognitive functioning. Estimates are reported as maximum likelihood (*est ML*) with 95% confidence interval and *P*-value. Significant *P*-values surviving Bonferroni correction for multiple testing (*P*<0.013) are marked in bold. EF = executive functioning

**Supplementary Table 9. Results of mediation analyses adjusting for white matter lesion volume**

|  | | Normalized volume |
| --- | --- | --- |
|  | | *est ML (95% confidence interval), P-value* |
| Average cognition | Direct (c)  Indirect 1 (a*b)  Indirect 2 (d*e)  Total (c+(a*b)+(d*e)) | *-0.01 (-0.06;0.04), P=0.699*  *-0.01 (-0.02;0.01), P=0.500*  *-0.04 (-0.06;-0.02), P=****0.001***  *-0.05 (-0.10;-0.00), P=0.046* |
| Information processing speed | Direct (c)  Indirect 1 (a*b)  Indirect 2 (d*e)  Total (c+(a*b)+(d*e)) | *-0.10 (-0.20;-0.00), P=0.049*  *0.03 (-0.00;0.05), P=0.066*  *-0.06 (-0.09;-0.02), P=****0.002***  *-0.13 (-0.23;-0.03), P=****0.009*** |
| Verbal memory | Direct (c)  Indirect 1 (a*b)  Indirect 2 (d*e)  Total (c+(a*b)+(d*e)) | *-0.05 (-0.14;0.05), P=0.354*  *-0.01 (-0.03;0.02), P=0.630*  *-0.06 (-0.09;-0.02), P=****0.002***  *-0.11 (-0.20;-0.02), P=0.020* |
| Visuospatial memory | Direct (c)  Indirect 1 (a*b)  Indirect 2 (d*e)  Total (c+(a*b)+(d*e)) | *0.04 (-0.11;0.18), P=0.607*  *0.00 (-0.03;0.04), P=0.837*  *-0.07 (-0.11;-0.02), P=****0.008***  *-0.02 (-0.16;0.11), P=0.730* |

Outcome variables (cognitive functioning at 5-year follow-up) are shown on the left, divided into four effect estimates: direct (c), indirect 1 (a*b), indirect 2 (d*e) and total effect (c+(a*b)+(d*e)). Predictor variable is log(x+1)-transformed cortical lesion volume, and mediator variables are the mean *Z*-scores of the normalized cortical volume in lesional cortex (indirect 1) and normal-appearing cortex (indirect 2). Included confounders are age, sex, high level of education, baseline cognitive functioning and log-transformed white matter lesion volume. Estimates are reported as maximum likelihood (*est ML*) with 95% confidence interval (CI) and *P*-value. Significant *P*-values surviving Bonferroni correction for multiple testing (*P*<0.013) are marked in bold. EF = executive functioning

**Supplementary Table 10. Results of mediation analyses within cortical networks**

|  | | Normalized volume |
| --- | --- | --- |
|  | | *est ML (95% confidence interval), P-value* |
| Average cognition | Direct (c)  Indirect  DAN (a*b)  DMN (d*e)  Limbic (f*g)  SMN (h*i)  VAN (j*k)  Total | *-0.03 (-0.08;0.03), P=0.360*  *-0.00 (-0.02;0.01), P=0.625*  *-0.01 (-0.02;0.01), P=0.400*  *-0.00 (-0.02;0.01), P=0.776*  *-0.03 (-0.04;-0.01), P=****0.004***  *-0.01 (-0.02;0.00), P=0.157*  *-0.07 (-0.11;-0.02), P=****0.003*** |
| EF – Inhibition | Direct (c)  Indirect  DAN (a*b)  DMN (d*e)  Limbic (f*g)  SMN (h*i)  VAN (j*k)  Total | *-0.09 (-0.25;0.08), P=0.313*  *0.06 (0.01;0.12), P=0.017*  *-0.07 (-0.12;-0.02), P=0.011*  *0.02 (-0.03;0.06), P=0.473*  *-0.01 (-0.05;0.04), P=0.765*  *-0.04 (-0.08;0.00), P=0.052*  *-0.12 (-0.27;0.03), P=0.114* |
| Information processing speed | Direct (c)  Indirect  DAN (a*b)  DMN (d*e)  Limbic (f*g)  SMN (h*i)  VAN (j*k)  Total | *-0.09 (-0.19;0.02), P=0.097*  *-0.02 (-0.05;0.01), P=0.171*  *-0.00 (-0.03;0.03), P=0.964*  *-0.02 (-0.05;0.01), P=0.172*  *0.03 (0.00;0.06), P=0.036*  *-0.02 (-0.04;0.00), P=0.085*  *-0.11 (-0.20;-0.02), P=0.013* |
| Verbal memory | Direct (c)  Indirect  DAN (a*b)  DMN (d*e)  Limbic (f*g)  SMN (h*i)  VAN (j*k)  Total | *-0.05 (-0.14;0.05), P=0.369*  *0.01 (-0.02;0.03), P=0.636*  *-0.02 (-0.04;0.01), P=0.171*  *-0.02 (-0.04;0.01), P=0.300*  *-0.04 (-0.07;-0.01), P=****0.010***  *-0.00 (-0.02;0.02), P=0.847*  *-0.12 (-0.20;-0.03), P=****0.006*** |
| Visuospatial memory | Direct (c)  Indirect  DAN (a*b)  DMN (d*e)  Limbic (f*g)  SMN (h*i)  VAN (j*k)  Total | *-0.05 (-0.19;0.11), P=0.560*  *0.03 (-0.02;0.07), P=0.235*  *-0.05 (-0.10;-0.01), P=0.021*  *-0.04 (-0.08;0.01), P=0.108*  *-0.07 (-0.12;-0.02), P=****0.008***  *0.02 (-0.01;0.05), P=0.161*  *-0.16 (-0.29;-0.03), P=0.019* |

Outcome variables (cognitive functioning at 5-year follow-up) are shown on the left, divided into seven effect estimates: direct (c), five indirect (e.g., a*b) and total effects (c+(a*b)+(d*e)+(f*g) +(h*i)+(j*k)). Predictor variable is log(x+1)-transformed total cortical lesion volume, and mediator variables are the mean *Z*-scores of normalized network volume of normal-appearing cortex. Included confounders are age, sex, high level of education, and baseline cognitive functioning. Estimates are reported as maximum likelihood (*est ML*) with 95% confidence interval and *P*-value. Significant *P*-values surviving Bonferroni correction for multiple testing (*P*<0.010) are marked in bold. EF = executive functioning; DAN = dorsal attention network; DMN = default mode network; SMN = sensorimotor network; VAN = ventral attention network.

**Supplementary Figure 1. Examples of cortical lesions identified in multiple sclerosis**


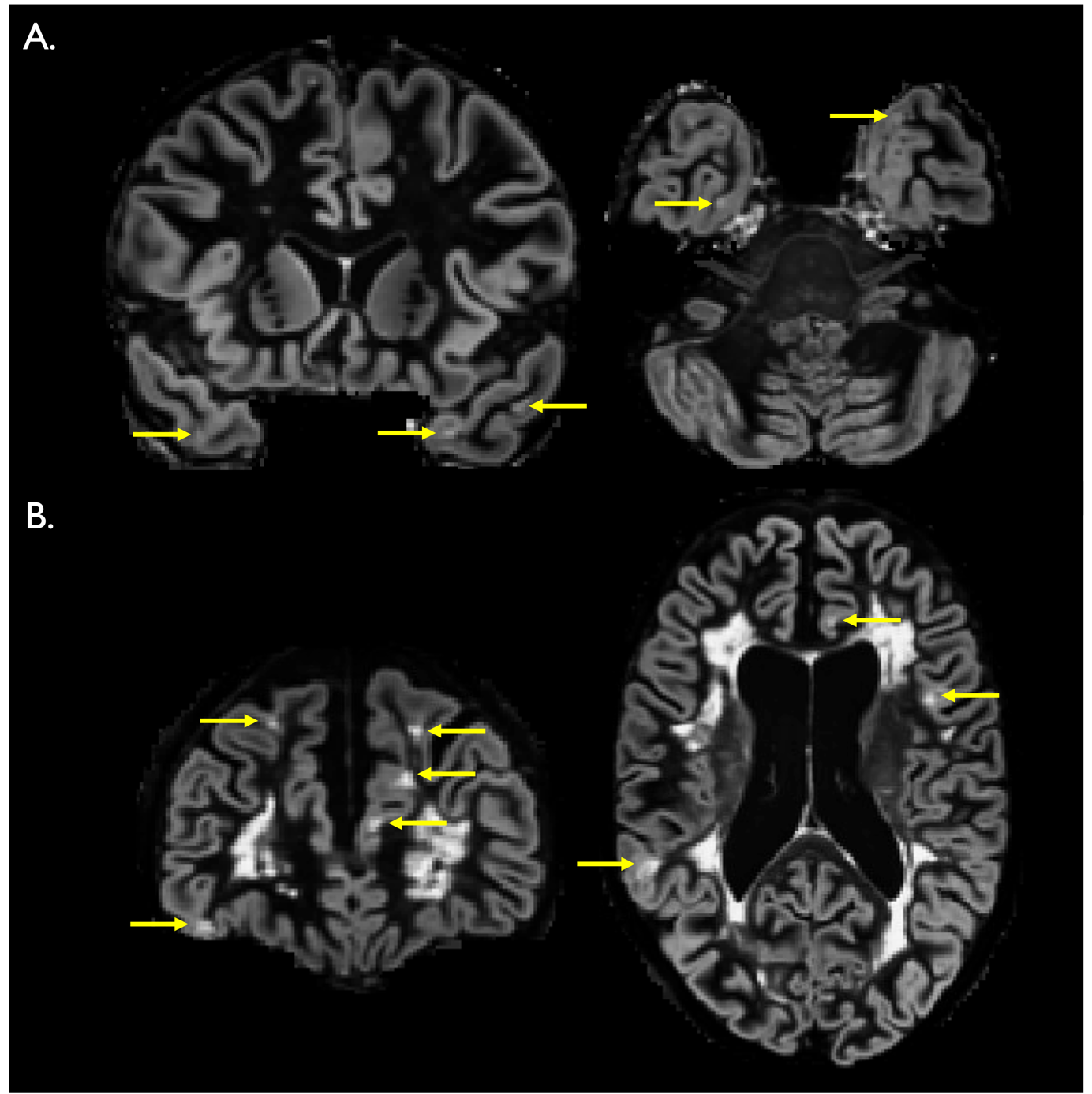


Coronal and axial slices of artificially-generated double inversion-recovery images of representative individuals with multiple sclerosis are displayed at the level of temporal cortical lesions (panel A) and of frontal/parietal cortical lesions (panel B). Yellow arrows point to the cortical lesions.
